# *srdA* mutations suppress the *rseA*/*cpsA* deletion mutant conidiation defect in *Aspergillus nidulans*

**DOI:** 10.1038/s41598-023-31363-8

**Published:** 2023-03-15

**Authors:** Masahiro Ogawa, Ryouichi Fukuda, Ryo Iwama, Yasuji Koyama, Hiroyuki Horiuchi

**Affiliations:** 1 Department of Biotechnology, The University of Tokyo, 1-1-1 Yayoi, Bunkyo-ku, Tokyo, 113-8657 Japan; 2 Noda Institute for Scientific Research, 338 Noda, Noda, Chiba 278-0037 Japan; 3 Collaborative Research Institute for Innovative Microbiology, The University of Tokyo, Yayoi 1-1-1, Bunkyo-ku, Tokyo, 113-8657 Japan

**Keywords:** Microbiology, Molecular biology

## Abstract

Conidiation is an important reproductive process in *Aspergillus*. We previously reported, in *A. nidulans,* that the deletion of a putative glycosyltransferase gene, *rseA/cpsA*, causes an increase in the production of extracellular hydrolases and a severe reduction in conidiation. The aim of this study was to obtain novel genetic factors involved in the repression of conidiation in the *rseA* deletion mutant. We isolated mutants in which the *rseA* deletion mutant conidiation defect is suppressed and performed a comparative genomic analysis of these mutants. A gene encoding a putative transcription factor was identified as the associated candidate causative gene. The candidate gene was designated as *srdA* (*s*uppressor gene for the conidiation defect of the r*seA* *d*eletion mutant). The conidiation efficiency of the *rseAsrdA* double-deletion mutant was increased. Introduction of wild-type *srdA* into the suppressor mutants caused a conidiation defect similar to that of the *rseA* deletion mutant. Notably, the conidiation efficiencies of the *rseAsrdA* double-deletion and *srdA* single-deletion mutants were higher than that of the wild-type strain. These results indicate that *srdA* is a novel genetic factor that strongly represses conidiation of the *rseA* deletion mutant, and a putative transcriptional regulator, SrdA is a negative regulator of conidiation in *A. nidulans*.

## Introduction

Filamentous fungi are known for their ability to secrete a wide variety of extracellular enzymes^1^. *Aspergillus* species, common filamentous fungi found in diverse environments, are distributed worldwide. Some *Aspergillus* species have been used widely in industrial biotechnology. Koji-molds, including *Aspergillus oryzae*, *Aspergillus sojae*, and *Aspergillus luchuensis*, have been used in traditional fermented food production in East Asia^2^. Meanwhile, *A. nidulans* has been used as a model organism for the study of molecular genetics in filamentous fungi^3^, and the regulatory mechanisms for the production of extracellular hydrolases (amylolytic and cellulolytic enzymes)^4,5^. The regulation of conidiation has also been studied in *A. nidulans*, and a large number of genes are known to be involved in the formation and maturation of conidia^6–8^.

The production of extracellular enzymes, using *Aspergillus*, is performed under solid-state cultivation (SSC) and liquid cultivation (LC). Production of extracellular enzymes in *Aspergillus* species is highly promoted under SSC, but less stimulated under LC^9,10^. During the fermentation process, fungi cells undergo fermentation-specific environmental stresses. The stresses under the SSC could lead to promote extracellular enzyme production. Therefore, fungal stress response is important for enzyme production. Intracellular signaling pathways, such as the high osmolality glycerol (HOG) and the cell wall integrity (CWI) pathways, have been reported to play important roles in yeast (*Saccharomyces cerevisiae*) stress response^11,12^. The *A. nidulans* HOG and CWI pathways have also been investigated^13,14^.

Previously, we have reported that the deletion of a putative glycosyltransferase gene*, rseA*/*cpsA,* in *A. nidulans* under SSC, causes an increase in the production of extracellular hydrolases^15^. We showed that the HOG pathway was involved in the elevated production of hydrolases, and the *rseA* deletion mutant displayed a severe conidiation defect^15^. However, the mechanism of how *rseA* deletion causes substantial reduction of conidiation remains unclear.

In this study, we aimed to obtain novel genetic factors involved in the repression of conidiation in the *rseA* deletion mutant. We isolated mutants in which the conidiation defect in the *rseA* deletion mutant is suppressed, then we identified the suppressor mutant-associated causative gene. Additionally, we constructed and characterized the double deletion mutant of *rseA* and the causative gene.

## Results

### Isolation and characterization of suppressor mutants

Previously, we observed a severe conidiation defect in the *rseA* deletion mutant^15^. In the present study, we isolated and characterized suppressor mutants, that is, mutant strains in which the conidiation defect of the *rseA* deletion was suppressed. Spontaneous mutants with a green color occurred in the reddish-brown colonies of the *rseA* deletion mutant (DRA), when grown on MMGp agar plates (Fig. S1A). This suggests that the conidiation defect in DRA was largely restored in these spontaneous mutants. As shown in Fig. S1A, mutants B and C were observed in one DRA colony (left panel), and mutant D was observed in another DRA colony (right panel). We isolated the conidia from mutants B and D for further analysis. The strains isolated from mutants B and D were designated as SMGC-1 and SMGC-2, respectively.

**Figure 1:**
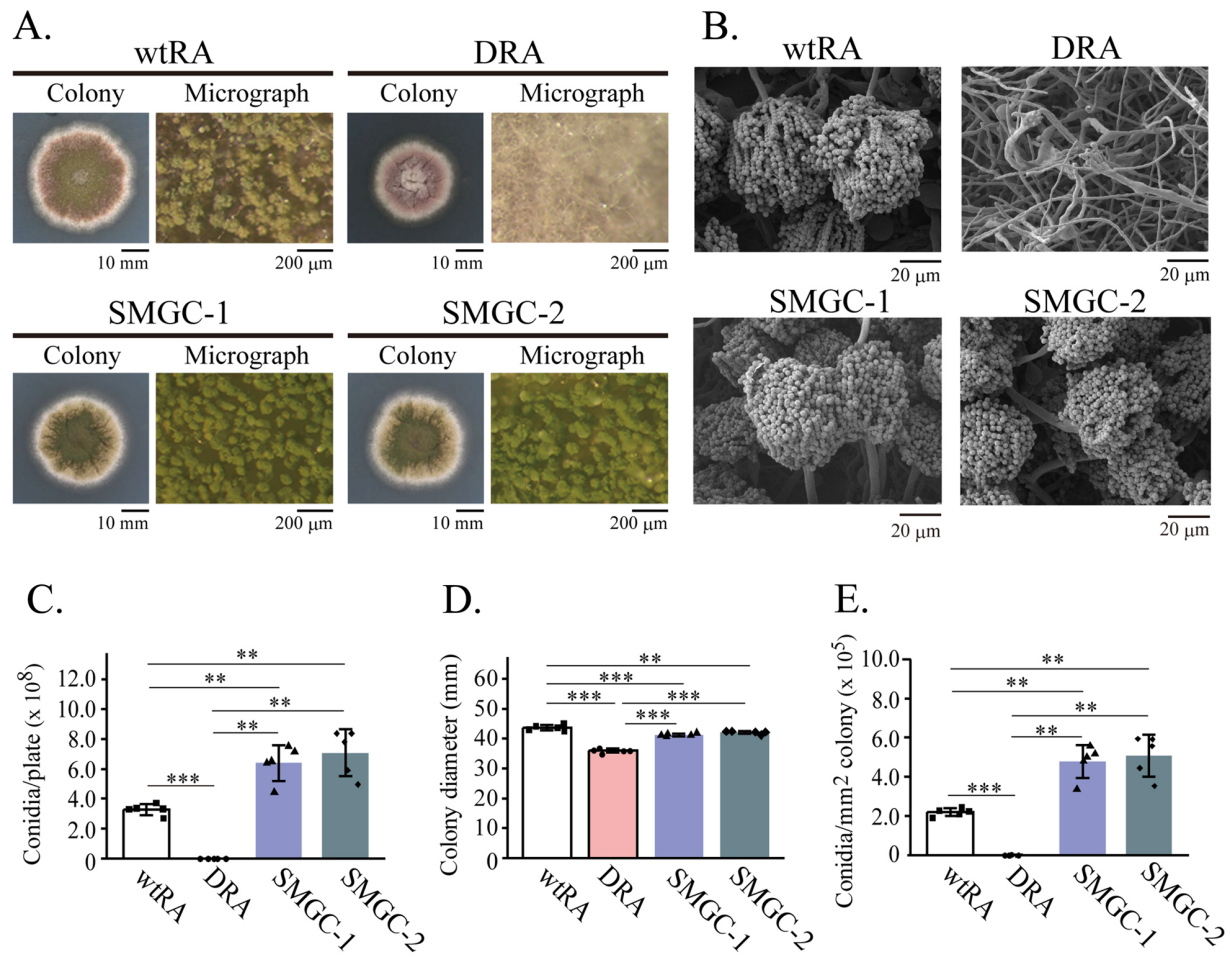
Characterization of the Δ*rseA* mutant (DRA), SMGC-1, and SMGC-2. (**A**) Colony growth appearances of DRA, SMGC-1, SMGC-2, and the wild type strain (wtRA) on MMGp agar plates (incubated at 37 °C for 5 days). (**B**) Scanning electron micrographs of the mutant (DRA, SMGC-1, SMGC-2) and wtRA conidiophores. (**C**) The mutant and wtRA conidiation efficiencies (Conidia/plate × 10^8^). (**D**) Average diameter (mm) of the mutant and wtRA colonies. (**E**) Number of conidia per mm^2^ of the mutant and wtRA colonies (Conidia/mm^2^ colony × 10^5^). Bars indicates standard deviations. *: *p* < 0.05, **: *p* < 0.01, and ***: *p* < 0.001 (Welch’s *t* test, *p*-values were adjusted for multiple comparison using holm’s method).

SMGC-1 and SMGC-2 formed colonies with appearances similar to that of the wild-type strain (wtRA; Fig. 1A). In DRA, conidiophores were scarcely observed and the conidiophore’s morphologies were aberrant. In contrast, the morphologies of SMGC-1 and SMGC-2 conidiophores were similar to that of wtRA (Fig. 1B).

The conidiation efficiency of DRA was drastically decreased. However, the conidiation efficiencies of SMGC-1 and -2 were approximately twofold higher than that of wtRA, indicating that conidiation was restored (Fig. 1C). Compared to wtRA, the colony size of DRA was reduced to 82.6% ± 1.5. Colony growth improvement was observed in SMGC-1 and -2. Relative to wtRA, SMGC-1 and -2 colony growth was 95.4% ± 1.0 and 96.4% ± 1.1, respectively (Fig. 1D). The number of conidia per mm^2^ in the DRA colony was 0.47% ± 0.27 of that of wtRA (Fig. 1E). Conversely, the number of conidia observed per mm^2^ in SMGC-1 and -2 was significantly greater than that of the wtRA (219% ± 38.8 and 232% ± 48.3, respectively) (Fig. 1E). Using Southern hybridization, we confirmed the deletion of *rseA* in the SMGC-1 and -2 (Fig. S1B). These results confirm that SMGC-1 and -2 are suppressor mutants of the DRA conidiation defect and are not revertants of the *rseA* deletion.

### Comparative genomic analysis of the suppressor mutants and the Δ*rseA* mutant

**Figure 2:**
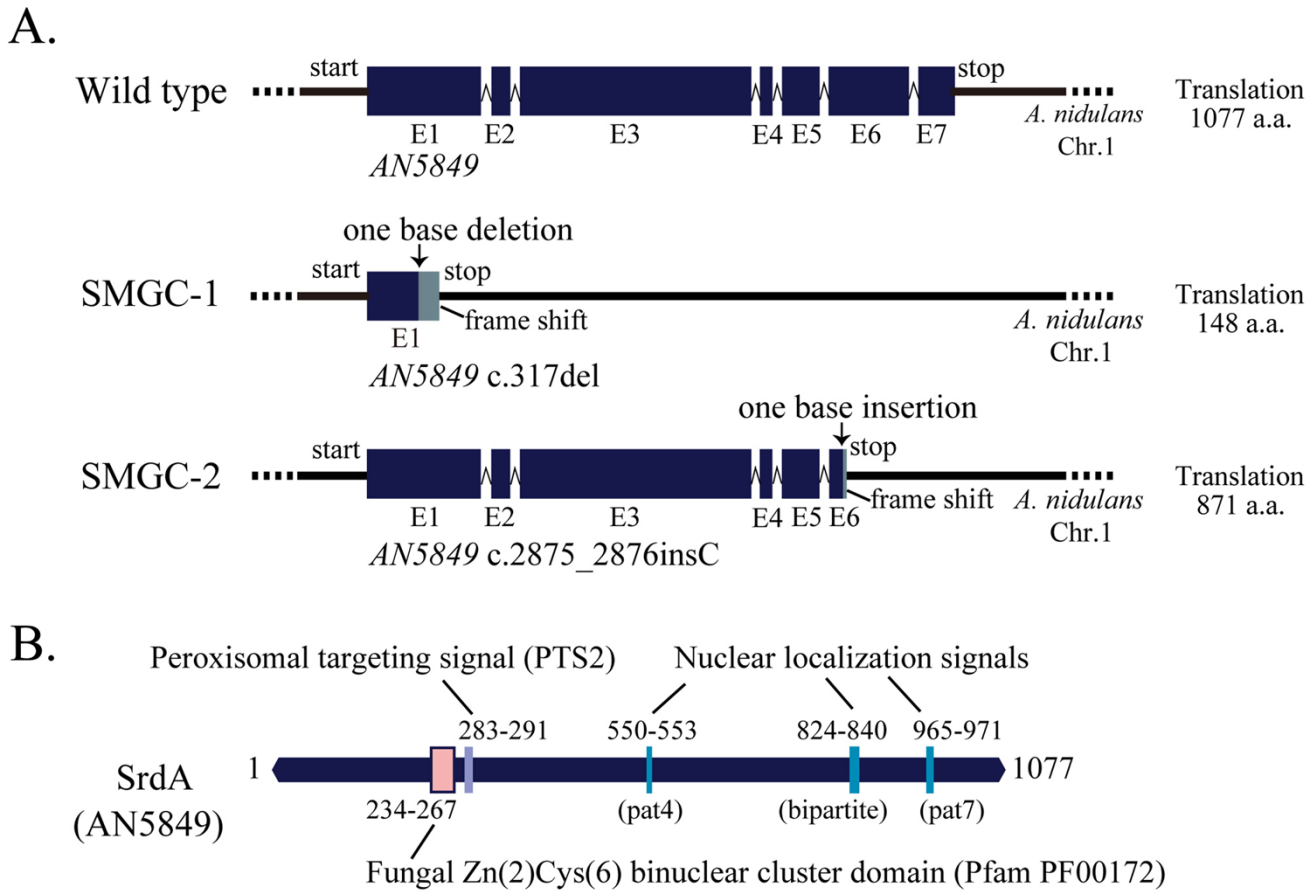
Positions of mutations in the *srdA* gene and gene product domain organization. (**A**) Gene structures of *srdA* (*AN5849*) in the wild-type strain, SMGC-1, and SMGC-2. Positions of exons, introns, and mutation points of *srdA* in SMGC-1, and SMGC-2 are indicated. (**B**) Domain organization of SrdA. Positions of a fungal Zn_2_Cys_6_ binuclear domain, nuclear localization signal motifs (NLSs), and a type 2 peroxisomal localization signal motif (PTS2).

To identify the causative gene(s) for the suppressor mutations in SMGC-1 and -2, we performed whole-genome sequencing. In the genome sequence of SMGC-1, two deletion mutations and two single-nucleotide substitutions were observed (Table 1A). In SMGC-2, one insertion and two single-nucleotide replacements were detected (Table 1B). Notably, mutations in *AN5849* have been found in both SMGC-1 and -2 genome sequences. The gene structures of *AN5849* in the wild-type and suppressor mutants are shown in Fig. 2A. In the wild-type strain, the open reading frame of *AN5849* consists of 3571 bp with seven exons (determined by RNA-seq analyses) (FungiDB: https://fungidb.org/fungidb/app). One base deletion in exon 1 and one base insertion in exon 6 of *AN5849* was detected in SMGC-1 and -2, respectively. The one-base alterations (deletion and insertion) result in frameshifts and cause pre-terminations. Therefore, *AN5849* is one of the candidate genes responsible for suppressor mutations.

**Table 1:** Mutations found in SMGC-1 and SMGC-2 compared to DRA (the parental strain) by NGS.

Chromosome number	Position	Gene ID	Exon number	Sequence of mutation site		Mutation type
				DRA (parent)	SMGC-1	
A. Mutations found in SMGC-1						
Chromosome I	2037572	AN5849	Exon 1	TGGGGGG	TGGGGG	Deletion
Chromosome VII	107165	–	–	CTGATGAT GATGATGA TGATG	CTGATGATGATGATG	Deletion
Chromosome VII	856427	tN(GUU)6	Exon 1	C	G	Replacement
Chromosome VIII	2936237	AN0633	Exon 2	G	A	Replacement

Chromosome number	Position	Gene ID	Exon number	Sequence of mutation site		Mutation type
				DRA (parent)	SMGC-2	
B. Mutations found in SMGC-2						
Chromosome I	810803	–	–	G	A	Replacement
Chromosome I	2035014	AN5849	Exon 6	TGGGGGG	TGGGGGGG	Insertion
Chromosome IV	2807567	–	–	C	T	Replacement

*AN5849* encodes a putative Zn_2_Cys_6_ transcription factor. Zn_2_Cys_6_ transcription factors are unique to fungi. Fifty-five Zn_2_Cys_6_ transcription factors, Gal4, Hap1, and Leu3, have been found in *S. cerevisiae*^16^. Some Zn_2_Cys_6_ transcription factors in *A. nidulans* have been characterized (*e.g.*, AlcA, PrnA, AflR, NirA, SclB, and McrA)^17–22^. However, the function of AN5849 has not been investigated. The domain structure of AN5849, based on the results of Pfam and WoLF-PSORT searches is shown in Fig. 2B^23,24^. AN5849 consists of 1077 amino acids, and a fungal Zn_2_Cys_6_ binuclear cluster domain motif is located at the N-terminal region (amino acid residues 234 to 267). Three nuclear localization signal (NLS) motifs and one peroxisome targeting signal (PTS2) motif have also been found in AN5849^23,25^. We designated *AN5849 srdA* as the *s*uppressor gene for the conidiation defect of the r*seA* *d*eletion mutant.

### Conidiation of the Δ*rseA*Δ*srdA *and Δ*srdA* mutants

**Figure 3:**
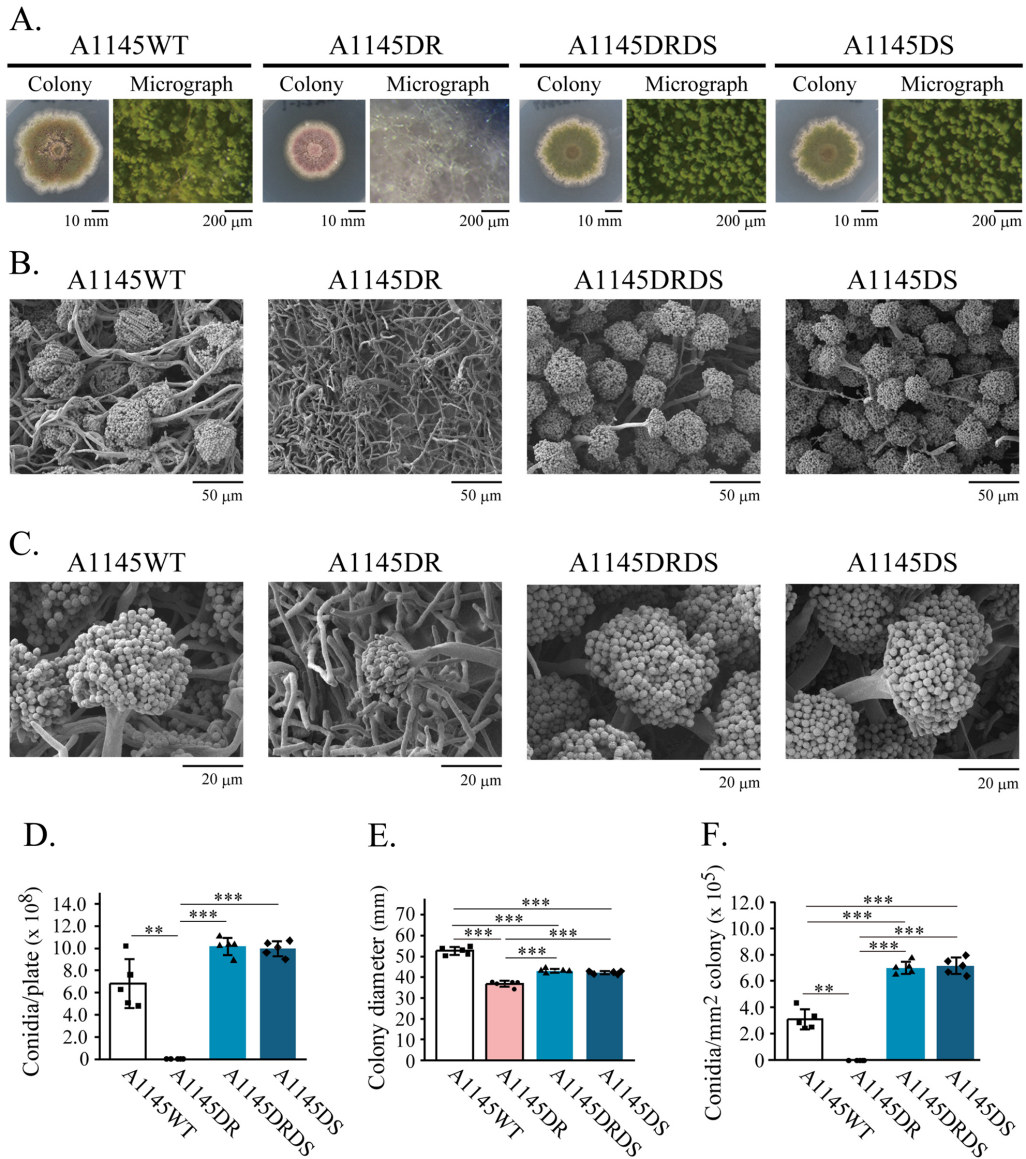
Characterizations of the Δ*rseΑ* (A1145DR), the Δ*rseA*Δ*srdA* (A1145DRDS), and the Δ*srdA* (A1145DS) mutants. **(A**) Colony growth appearances of A1145DR, A1145DRDS, A1145DS, and A1145WT on MMGp agar plates (incubated at 37 °C for 5 days). (**B**) Scanning electron micrographs of A1145DR, A1145DRDS, and A1145DS. (**C**) Scanning electron micrographs of A1145DR, A1145DRDS, and A1145DS at higher magnifications. (**D**) Conidiation efficiencies. (**E**) Average diameter of the colonies. (**F**) The number of conidia per mm^2^ colony of the samples. Bars indicate standard deviations. *: *p* < 0.05, **: *p* < 0.01, and ***: *p* < 0.001 (Welch’s *t* test, *p*-values were adjusted for multiple comparison using Holm's method).

**Figure 4:**
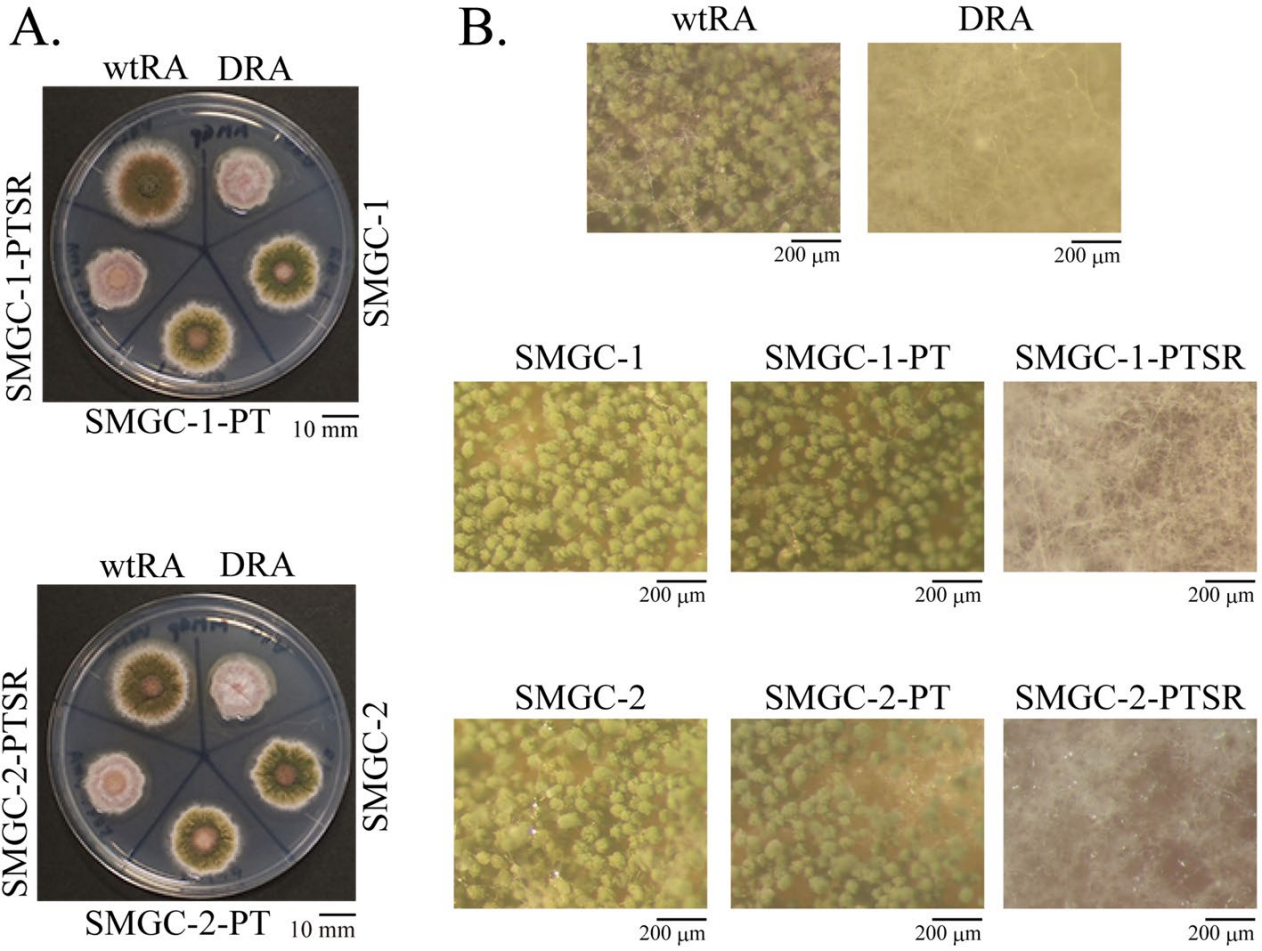
Introduction of wild type *srdA* into SMGC-1 and SMGC-2. (**A**) Colonies of the wild-type strain (wtRA), the Δ*rseA* mutant (DRA), the suppressor mutants (SMGC-1 and SMGC-2), the control strains in which only a *ptrA* marker gene was introduced (SMGC-1-PT and SMGC-2-PT), and the strains in which both *ptrA* and *srdA* were introduced (SMGC-1-PTSR and SMGC-2-PTSR). (**B**) Stereomicroscope observations of colony centers. 5 × 10^4^ of conidia of these strains were inoculated on MMGp agar plates and incubated at 37 °C for 3 days.

To determine whether *srdA* was the causative gene for suppressor mutations of the Δ*rseA* mutant conidiation defect, we constructed and characterized a Δ*rseA*Δ*srdA* mutant (A1145DRDS) and the corresponding Δ*rseA* mutant (A1145DR). Colony appearances and SEM micrographs of these strains are shown in Fig. 3A–C. Similar to DRA, defects in conidiophore formation were observed in A1145DR. In contrast, the morphologies of the A1145DRDS conidiophores were similar to that of the wild-type (A1145WT). The conidiation efficiency of A1145DR was low (Fig. 3D). However, the conidiation efficiency of A1145DRDS was higher than that of A1145WT (Fig. 3D). Compared to the wild-type, the colony diameters of A1145DR and A1145DRDS significantly decreased, whereas the colony diameter of A1145DRDS was larger than that of A1145DR (Fig. 3E). Compared to the wild-type, the number of conidia per mm^2^ in the A1145DR colony decreased (0.37% ± 0.24). In contrast, that of A1145DRDS increased (227% ± 15.3) (Fig. 3F). These A1145DRDS phenotypes are similar to those of SMGC-1 and -2 (Fig. 1). We further examined *srdA* as the causative gene for suppressor mutations, by introducing wild-type *srdA* into SMGC-1 and -2 (Fig. 4). The *srdA*-introduced strains SMGC-1-PTSR and SMGC-2-PTSR displayed conidiation defects similar to that observed in DRA. Therefore, we concluded that the mutations on *srdA* observed in SMGC-1 and SMGC-2 are responsible for suppression of conidiation defects in the *rseA* deletion mutant.

We also constructed the Δ*srdA* mutant, A1145DS. The colony appearance of A1145DS was similar to that of A1145WT and A1145DRDS. The conidiation efficiency, colony diameter, number of conidia per mm^2^ colony, and conidiophore morphology of A1145DS were all similar to that of A1145DRDS (Fig. 3). These results indicate that the *srdA* mutation is epistatic to the *rseA* mutation with regard to *A. nidulans* conidiation and growth.

### Extracellular enzyme production of the Δ*rseA*Δ*srdA* mutant

**Figure 5:**
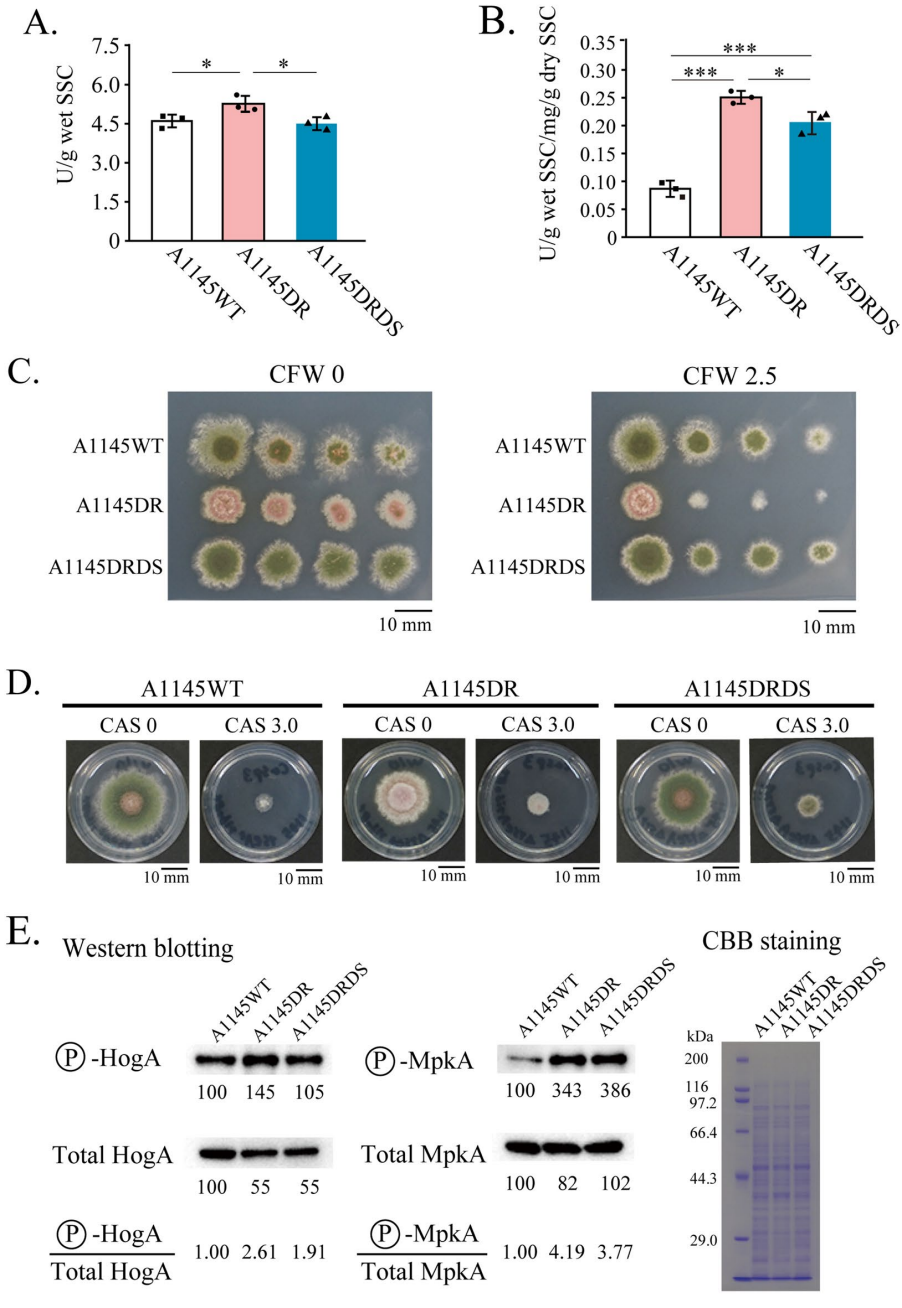
Extracellular endo-xylanase production, sensitivities to cell wall perturbating agents, and the phosphorylation statuses of MAP kinases in the Δ*rseA* mutant (A1145DR), the Δ*rseA*Δ*srdA* mutant (A1145DRDS), and the wild type strain (A1145WT). (**A**) Endo-xylanase production under the solid-state cultivation condition. (**B**) Endo-xylanase production per mg mycelia. Experiments were conducted in triplicate. Bars indicate standard deviations. *: *p* < 0.05, **: *p* < 0.01, and ***: *p* < 0.001 (student’s *t* test, *p*-values were adjusted for multiple comparison using holm’s method). (**C**) Growth sensitivities to calcofluor white (CFW). The concentrations in the medium are shown to the right of CFW (μg/mL). (**D**) Growth sensitivities to caspofungin (CAS). The concentrations in the medium are shown to the right of CAS (μg/mL). (**E**) Phosphorylation statuses of HogA and MpkA. Relative signal intensities of western blot bands are indicated. Signal intensity of A1145WT was defined as 100. Ratios of phosphorylated HogA per total HogA, and those of phosphorylated MpkA per total MpkA are also visible. CBB staining of the PAGE samples were used as loading control. The original data of the western bolts and SDS-PAGE are shown in Figs. S7 to S11.

We previously reported that extracellular hydrolase production increased in the Δ*rseA* mutant under a solid-state cultivation condition^15^. To determine whether the *srdA* deletion contributes to the increased extracellular hydrolase production of the Δ*rseA* mutant, we examined the extracellular endo-xylanase production by A1145DRDS (Fig. 5A). Endo-xylanase activity per g solid-state culture (SSC) was increased in A1145DR (5.26 ± 0.31 U/g wet SSC) compared to that of A1145WT (4.60 ± 0.24 U/g wet SSC) and decreased in A1145DRDS (4.50 ± 0.25 U/g wet SSC). To evaluate the growth of the fungal strains under SSC conditions, we estimated mycelial mass, by the chitin content in the dry SSC of the strains (A1145WT, 3,300 ± 703 μg/g; A1145DR, 1,706 ± 125 μg/g; A1145DRDS, 1,766 ± 124 μg/g) and those in the dry mycelia from liquid culture (A1145WT, 60.6 μg/mg; A1145DR, 81.1 μg/mg; A1145DRDS, 79.9 μg/mg). The masses of A1145WT, A1145DR, and A1145DRDS in the dry SSC were estimated at 54.4 ± 11.6 mg/g, 21.0 ± 1.5 mg/g, and 22.1 ± 1.6 mg/g, respectively. Therefore, the mycelial masses of A1145DR and A1145DRDS grown under SSC conditions were less than that of A1145WT. The extracellular endo-xylanase activities per mg of mycelia of A1145WT, A1145DR, and A1145DRDS were 0.087 ± 0.015 U/g wet SSC/mg/g dry SSC, 0.251 ± 0.012 U/g wet SSC/mg/g dry SSC, and 0.205 ± 0.020 U/g wet SSC/mg/g dry SSC, respectively (Fig. 5B). These results showed that the capacity of extracellular endo-xylanase production by A1145DRDS was significantly higher than that of A1145WT, with a capacity similar to that of A1145 DR. This finding suggests that the *srdA* deletion does not significantly affect the Δ*rseA* mutant ability to produce extracellular endo-xylanase.

### Growth sensitivity of the Δ*rseA*Δ*srdA* mutant to cell wall perturbating agents

Since the Δ*rseA* mutant of *A. nidulans* was highly sensitive to calcofluor white (CFW) and resistant to caspofungin (CAS)^15,26^, we examined the growth sensitivity of A1145DRDS to these cell wall-perturbating agents (Fig. 5C,D). While A1145DR was more sensitive to CFW than A1145WT, the sensitivity of A1145DRDS was similar to that of A1145WT. In contrast, compared to A1145WT, A1145DRDS was resistant to CAS, which was similar to that of A1145 DR. These results suggest that the cell wall architecture of the Δ*rseA*Δ*srdA* mutant is partially different from that of the Δ*rseA* mutant.

### Activation state of intracellular signaling pathways in the Δ*rseA*Δ*srdA* mutant

We previously reported that intracellular signaling pathways, such as the HOG and CWI pathways, were highly activated in the *rseA* deletion mutant^15^. To evaluate the effects of *srdA* deletion on the activation of these pathways, in the Δ*rseA* mutant, we determined the phosphorylation states of HogA and MpkA, the mitogen activated protein (MAP) kinases in the HOG and CWI pathways, in A1145DRDS (Fig. 5E). The signal intensity of phosphorylated HogA increased in A1145DR compared to that in A1145WT. However, phosphorylated HogA in A1145DRDS was similar to that of A1145WT. The signal intensity of total HogA decreased in A1145DR and A1145DRDS*.* Therefore, the ratio of phosphorylated HogA to total HogA increased in both A1145DR and A1145DRDS. Similarly, the ratio of phosphorylated MpkA to total MpkA was higher in A1145DR and A1145DRDS (Fig. 5E). However, these ratios in A1145DRDS were lower than that in A1145DR. These results indicate that HOG and CWI pathway are activated in the Δ*rseA*Δ*srdA* mutant. However, activation levels are attenuated by the *srdA* deletion.

### Production of the extracellular endo-xylanase and the activation of the HOG pathway in the Δ*srdA* mutant

The production of endo-xylanase in A1145DS was measured under SSC conditions (Fig. S2A,B). Endo-xylanase activity per g SSC of A1145DS was lower than that of A1145WT (4.79 ± 0.09 U/g wet SSC vs 6.15 ± 0.56 U/g wet SSC). To evaluate the growth of these strains under SSC conditions, we estimated mycelial mass, by the chitin content in the dry SSC of the strains (A1145DS, 2437 ± 113 μg/g; A1145WT, 4629 ± 321 μg/g) and those in the dry mycelia from liquid culture (A1145DS, 72.0 μg/mg; A1145WT, 60.6 μg/mg). The mycelial mass of A1145DS was significantly lower than that of A1145WT (33.8 ± 1.6 mg/g dry SSC vs 76.4 ± 5.3 mg/g dry SSC). Consequently, the activity per mg mycelia (U/g wet SSC/mg/g dry SSC) of A1145DS increased (0.142 ± 0.006) compared to that of A1145WT (0.80 ± 0.05) (Fig. S2B)*.* These results indicate that the *srdA* deletion induced a modestly higher production of extracellular endo-xylanase in *A. nidulans*. The ratio of the phosphorylated HogA to total HogA was decreased in A1145 DS (Fig. S2C). The results indicate that, *srdA* deletion did not activate the HOG pathway in the wild-type strain.

### Presence of SrdA orthologs in 23 species of filamentous fungi

**Figure 6:**
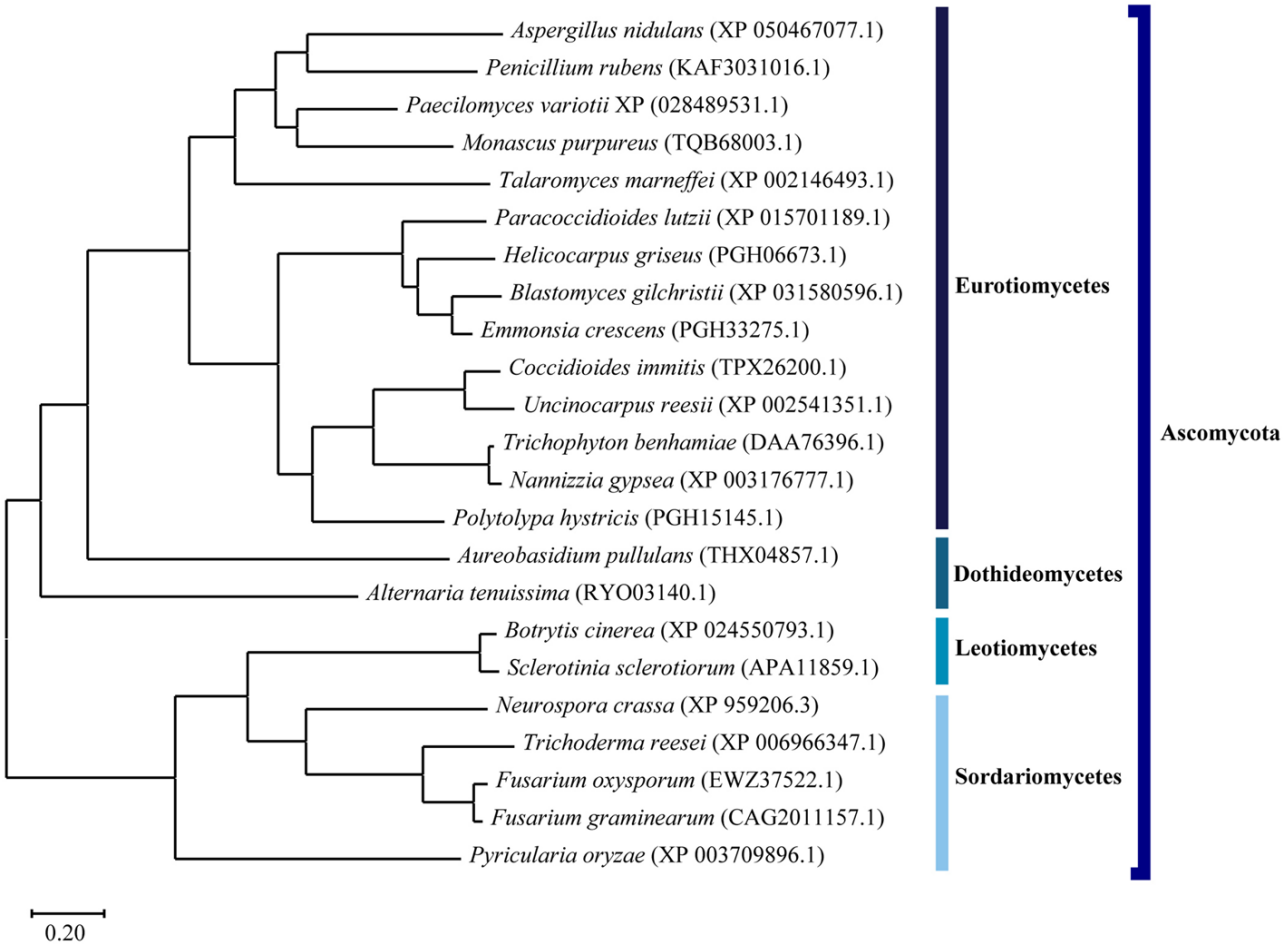
A phylogenetic tree of SrdA orthologs in the 23 filamentous fungi. NCBI RefSeq accession numbers or GenBank accession numbers of the SrdA orthologs are indicated in parentheses.

We searched for SrdA orthologs in 23 filamentous fungi, belonging to Ascomycota, and constructed a phylogenetic tree of the SrdA orthologs using MEGA11 (Fig. 6)^27^. Amino acid sequences of the SrdA orthologs were aligned using the Clustal W algorithm^28^. The taxonomy of each fungal species is shown according to the NCBI Taxonomy browser^29^. The amino acid identity among the SrdA orthologs is indicated in Fig. S3. SrdA orthologs were conserved among Eurotiomycetes (> 38% amino acid identity) and in Dothideomycetes, Leotiomycetes, and Sordariomycetes (SrdA orthologs amino acid identity ranged between 25 and 32%). *rseA*/*cpsA* orthologs have been characterized in *A. nidulans*, *A. fumigatus, Pyricularia oryzae,* and *Neurospora crassa*^15,26,30–32^. SrdA orthologs were also identified in these fungal strains. In addition, the 23 filamentous fungi described above possessed RseA/CpsA orthologs (Fig. S4). RseA/CpsA orthologs are distributed in Basidiomycota (Agaricomycetes and Tremellomycetes) and Mucoromycota (Mucoromycetes). However, no SrdA orthologs have been identified in Basidiomycota and Mucoromycota. Furthermore, the budding yeast *S. cerevisiae,* fission yeast *Schizosaccharomyces pombe*; and dimorphic yeasts *Candida albicans* and *Yarrowia lipolytica* do not possess Rse/CpsA nor SrdA orthologs.

## Discussion

Conidiation and noteworthy capacity for production of extracellular enzymes are important factors that drive the industrial utilization of filamentous fungi. The deletion of *rseA* causes hyper-production of extracellular enzymes in *A. nidulans*, but results in a conidiation defect^15^. In this study, we identified the causative gene that suppresses the conidiation defect in the Δ*rseA* mutant. Conidiation of the Δ*rseA* mutant was restored by the *srdA* deletion (Fig. 3). This suggests that SrdA plays a major role in the repression of the conidiation of the Δ*rseA* mutant.*rseA* encodes a putative GT family 2 glycosyltransferase, which is homologous to a hyaluronic acid synthetase (Cps1) of *Cryptococcus neoformans*^33,34^. Deletion of *rseA/cpsA* affects hyphal growth and morphogenesis of *A. nidulans* and its secondary metabolite and extracellular enzyme production^15,26^. Recently, it was reported that *rseA*/*cpsA* orthologs influence plant and human pathogens^30,32,35^. Since deletion of *rseA* or its ortholog in *N. crassa* affects cell wall composition and causes hyperactivation of the CWI pathway, it has been proposed that RseA and its ortholog play a role in cell wall biogenesis^15,26,31^. Although the growth sensitivity of the Δ*rseA*Δ*srdA* mutant to CFW was similar to that of the wild-type strain, the mutant was resistant to CAS (Fig. 5C, Δ*rseA*Δ*srdA* CAS sensitivity is similar to that of Δ*rseA*). These results indicate that restoration of cell wall integrity in the Δ*rseA*Δ*srdA* mutant is limited. In addition, hyperactivation of the CWI pathway was observed which supports the previous statement. The HOG pathway was activated in the Δ*rseA*Δ*srdA* mutant as well as in the Δ*rseA* mutant (Fig. 5E). Since this pathway is known to be involved in maintaining the cell wall integrity of *A. nidulans*^36^, the results suggest that the observed HOG pathway activation in the Δ*rseA*Δ*srdA* mutant is due to perturbation of fungal cell wall integrity.

An increase in conidiation efficiency was observed in both the Δ*rseA*Δ*srdA* and Δ*srdA* mutants (Fig. 3). This suggests that SrdA is a negative regulator of *A. nidulans* conidiation. Moreover, we observed that the expression levels of *brlA*, *abaA*, and *wetA* were significantly increased in A1145DRDS compared to those in A1145DR and were higher in A1145DS than in A1145WT (our unpublished results). These data are consistent with our hypothesis that SrdA acts as a negative regulator of conidiation and functions upstream of BrlA. Many genes negatively affect conidiation in *A. nidulans*^8^. Among these genes, *nsdD* and *rocA* encode putative transcription factors, and their deletions cause hyper-conidiation on agar media^37,38^. NsdD and RocA are thought to function upstream of *brlA*, which is the first transcription factor to function in the central regulatory cascade of *A. nidulans* conidiation^6–8^. Furthermore, a putative Zn_2_Cys_6_ transcription factor, SfgA, acts as a negative regulator of conidiation^37,39^. In addition to these three negative regulators, SrdA may repress the conidiation of *A. nidulans*. Further investigation is required to clarify their functional relationships.

In the conidiation regulators of *A. nidulans*, CsgA, McrA, SclB, SfgA, and ZcfA are Zn_2_Cys_6_ transcription factors, as well as SrdA. SclB and McrA positively regulate conidiation, whereas CsgA and ZcfA are necessary for proper asexual and sexual development^21,22,40,41^. It has been reported that the heterodimer of the velvet proteins, VosA-VelB, directly binds to the promoter region of the target genes and regulates conidiation and spore viability^42–44^. The expression of *sclB* and *mcrA* are regulated by the VosA-VelB complex^21,22^. Although SrdA is not a positive regulator, VosA-VelB complex may be involved in regulating *srdA* expression. To date, the genes regulated by SrdA remain unknown. Transcriptome analysis of the Δ*rseA*Δ*srdA* mutant and Δ*srdA* mutant will provide useful information for understanding the biological functions of SrdA.

Two hundred and six of 1077 amino acid residues in the C-terminal region of SrdA were deleted in the SMGC-2 mutant. However, the phenotype was similar to that of the Δ*rseA*Δ*srdA* mutant (Fig. 3). This result indicated that the C-terminal of SrdA contains a region required for functional conidiation. Since an NLS is present in this region, it is possible that the mutant protein could not localize and therefore not function in the nuclei. It has been reported that the C-terminal domains of AmyR, a Zn_2_Cys_6_ transcription factor for amylolytic genes, are necessary for the regulation of subcellular localization and sensing the stimulation of inducers^45^. Therefore, it is suggested that the C-terminal region of SrdA is indispensable for sensing intracellular signal(s) that regulate localization and/or function.

Extracellular endo-xylanase production per milligram of mycelia was significantly increased in the Δ*rseA*Δ*srdA* mutant (Fig. 5B), and we observed that the HogA pathway was activated in this mutant (Fig. 5E). These results are consistent with our previous proposal, which suggests that the HOG pathway is involved in the increased production of extracellular hydrolases in the *rseA* deletion mutant^15^. Endo-xylanase production per milligram of mycelia was partially increased in the Δ*srdA* mutant, whereas the phosphorylation of HogA was remarkably attenuated, compared to the wild-type strain (Fig. S2B,C). Therefore, the mechanism of increased extracellular endo-xylanase production, caused by *srdA* deletion, is suggested to be independent of HOG pathway activation.

In this study, we indicated that loss of function of *srdA* caused suppression of conidiation defect in *rseA* deletion mutant. Moreover, SrdA function in conidiation regulation was analyzed. *srdA* orthologs are conserved in many filamentous fungi in Ascomycota. Therefore, it is expected that the reduced conidiation phenotypes of some koji*-*molds, caused by genetic manipulations for improvement in the production of enzymes and metabolites, could be suppressed by the deletion of *srdA* orthologs. Furthermore, we believe that the elucidation of SrdA function will contribute to understanding the developmental control of *Aspergillus* species.

Although *rseA*/*cpsA* is known to be involved in the regulation of secondary metabolite production, the effect of *srdA* mutations on secondary metabolite production in *rseA* deletion mutant has not been investigated in this study, because the fungal strains constructed in this study possess a mutation in *veA* (*veA1*). The gene, *veA*, plays crucial roles in regulating the secondary metabolite production^46^. Furthermore, it has also been reported that LaeA and velvet (VeA*)* family proteins coordinately regulate not only secondary metabolite production, but also the developments of *A. nidulans*^47^. Thus, under the *veA*^+^ background, the phenotype of Δ*srdA*-single deletion mutant and Δ*rseA*Δ*srdA*-double deletion mutant might be different from the phenotype of those observed in this study.

## Methods

### Fungal strains and media

The fungal strains used in this study and their origins are listed in Table 2. The fungal strains were cultured in YG complete medium^48^ and MMG minimal medium^49^. Pyridoxine-auxotrophic mutants were cultured in pyridoxine-supplemented MMG medium (MMGp; 0.5 μg/mL pyridoxine). Fungal strains possessing *pyrG89* and *riboB2* mutations were cultured in MMG medium supplemented with uracil (10 mM), uridine (10 mM), and riboflavin (2.5 μg/mL). Agar plates, used for fungi culture, were prepared by adding 1.5% agar to the culture media.

**Table 2:** *Aspergillus nidulans* strains used in this study.

Strain name	Genotype	Origin
A26	*biA1, veA1*	FGSC^a^
A1149	*pyrG89*; *pyroA4 nkuA*::*argB, veA1*	Reference^51^
A1145	*pyrG89*; *pyroA4 nkuA*::*argB*; *riboB2, veA1*	Reference^51^
DRA	*pyrG89*; *pyroA4 nkuA*::*argB*; *rseA(/cpsA)*::*pyrG, veA1*	Reference^15^
wtRA	*pyrG89*; *pyroA4 nkuA*::*argB*; *rseA*::*pyrG-rseA, veA1*	Reference^15^
SMGC-1	*srdA* c.317del *pyrG89*; *pyroA4 nkuA*::*argB*; *rseA*::*pyrG, veA1*	This study
SMGC-1-PT	*srdA* c.317del *pyrG89*; *pyroA4*::*pyroA4-ptrA nkuA*::*argB; rseA*::*pyrG, veA1*	This study
SMGC-1-PTSR	*srdA* c.317del *pyrG89; pyroA4*::*pyroA4-srdA-ptrA nkuA*::*argB; rseA*::*pyrG, veA1*	This study
SMGC-2	*srdA* c.2875_2876insC *pyrG89*; *pyroA4 nkuA*::*argB*; *rseA*::*pyrG, veA1*	This study
SMGC-2-PT	*srdA* c.2875_2876insC *pyrG89*; *pyroA4*::*pyroA4-ptrA nkuA*::*argB; rseA*::*pyrG, veA1*	This study
SMGC-2-PTSR	*srdA* c.2875_2876insC *pyrG89; pyroA4*::*pyroA4-srdA-ptrA nkuA*::*argB;rseA*::*pyrG, veA1*	This study
A1145-riboB2	*pyrG89*; *pyroA4 nkuA*::*argB*; *rseA*::*pyrG-rseA*; *riboB2, veA1*	This study
ΔrseA-riboB2	*pyrG89*; *pyroA4 nkuA*::*argB*; *rseA*::*pyrG*; *riboB2, veA1*	This study
A1145DRDS	*srdA*::*PgpdA-riboB* E217K*-TgpdA pyrG89*; *pyroA4*; *rseA*::*pyrG*; *riboB2, veA1*	This study
A1145DR	*pyrG89*; *pyroA4*::*pyroA4-PgpdA-riboB* E217K*-TgpdA nkuA*::*argB; rseA*::*pyrG*; *riboB2, veA1*	This study
A1145WT	*pyrG89*; *pyroA4*::*pyroA4-PgpdA-riboB* E217K*-TgpdA nkuA*::*argB*; *rseA*::*pyrG-rseA*; *riboB2, veA1*	This study
ΔsrdA-pyrG89	*srdA*::*PgpdA-riboB* E217K*-TgpdA pyrG89*; *pyroA4*; *riboB2, veA1*	This study
A1145DS	*srdA*::*PgpdA-riboB* E217K*-TgpdA pyrG89*; *pyroA4*; *rseA*::*pyrG-rseA*; *riboB2, veA1*	This study

^a^Fungal genetics stock center.

### General DNA techniques and fungal transformation

Oligonucleotide primers used in this study are listed in Table S1. High-fidelity enzymes, KOD plus Neo (Toyobo, Osaka, Japan) and PrimeSTAR MAX DNA polymerase (Takara Bio, Shiga, Japan), were used to amplify DNA fragments for plasmid construction and fungal transformation. The DNA polymerases were used according to the manufacturer’s instructions. The sequences of the DNA fragment inserts, contained in the plasmid DNA, were confirmed using standard Sanger sequencing (Eurofins Genomics K.K., Tokyo, Japan). Fungal transformations were performed using the protoplast-PEG method^50^. Southern hybridization was performed using a digoxigenin (DIG) labeling system (Roche Diagnostics, Basel, Switzerland) according to the instruction manual of Roche Diagnostics.

### Construction of Δ*rseA* deletion mutants with riboflavin auxotrophy

In *A. nidulans* A1145^51^, *rseA* was replaced with *pyrG* as previously described^15^. The obtained transformants were designated as ΔrseA-riboB2-1–3. The *rseA* deletions were confirmed by Southern hybridization (Fig. S5A).

### Construction of Δ*rseA*Δ*srdA* and Δ*srdA* mutants

The *gpdA* promoter, *riboB*, and *gpdA* terminator coding regions were amplified by PCR from *A. nidulans* A26 genomic DNA. The three amplified DNA fragments were ligated using fusion PCR^52^. The pUC-PTgpdA-riboB plasmid was generated by cloning the fusion PCR product into pUC118, using the SLiCE reaction^53^. A mutation, causing one amino acid exchange (E217K), was found in the *riboB* of pUC-PTgpdA-riboB. However, this mutation did not affect the intended function as a marker gene. Therefore, the plasmid was utilized in the present study.

A 5.9-kb DNA fragment containing *srdA* (*AN5849*) was amplified by PCR from the genomic DNA of *A. nidulans* A26. The PCR product was cloned into pUC118 to generate the pUC-AN5849 plasmid. The *riboB* marker cassette, amplified from pUC-PTgpdA-riboB, was introduced into pUC-AN5849 by the SLiCE reaction^53^ to generate the pUC-AN5849-DEL plasmid. A DNA fragment containing the *srdA* deletion was amplified by PCR from pUC-AN5849-DEL and introduced into *A. nidulans* ΔrseA-riboB2 and *A. nidulans* A1145 by the protoplast-PEG method^50^. The products, Δ*rseAΔsrdA* mutants (A1145DRDS-1, -2, and -3) and an Δ*srdA* mutant with uracil-auxotrophic properties (ΔsrdA-pyrG89), were obtained. *pyrG* was introduced into ΔsrdA-pyrG89 by the protoplast-PEG method, as previously described^15^. The *pyrG* complemented ΔsrdA-pyrG89 mutants were designated as A1145DS-1, -2, and -3 (*i.e.*, Δ*srdA* mutants). Southern hybridization was used to confirm the *srdA* deletion and *pyrG* introduction (Figs. S5B and S6A). As each of A1145DRDS-1–3 and each of A1145DS-1–3 showed the same phenotypes, we used A1145DRDS-1 and A1145DS-1 for further experiments as A1145DRDS and A1145DS, respectively.

### Introduction of *riboB* into ΔrseA-riboB2

A 4.2-kb DNA fragment, containing the intergenic region between *pyroA* and *sac3*, was amplified by PCR from the genomic DNA of *A. nidulans* A1145. The PCR product was cloned into pUC118 to generate the pUC-PSIG plasmid. The *riboB* marker cassette was then inserted into pUC-PSIG to obtain the pUC-PSIG-riboB plasmid. The DNA fragment for *riboB* complementation was prepared from pUC-PISG-riboB and introduced into *A. nidulans* ΔrseA-riboB2. The *riboB* complemented ΔrseA-riboB2 was designated A1145DR-1, -2, and -3 (*i.e.*, Δ*rseA* mutants). The *riboB* integrations into the A1145DR-1–3 genomes were confirmed by Southern hybridization (Fig. S6B). Since these three strains displayed the same phenotype, we used A1145DR-1 for further experiments as A1145DR.

### Introduction of *pyrG* and *riboB* into *A. nidulans* A1145

To obtain A1145-riboB2, *pyrG* was introduced into *A. nidulans* A1145, as previously described^15^ and designated as A1145-riboB2. The DNA fragment for *riboB* complementation was then introduced into A1145-riboB2. *riboB*-complemented A1145-riboB2 was designated as A1145WT-1, -2, and -3. Integrations of *pyrG* and *riboB* in the A1145WT-1–3 genomes were confirmed by Southern hybridization (Figs. S5B and S6B). We used A1145WT-1 for further experiments as A1145WT.

### Construction of SMGC-1 and SMGC-2-derived strains

A pyrithiamine-resistant marker (*ptrA*) was amplified from pPTR I (Takara Bio, Shiga, Japan) and inserted into pUC-PSIG to generate the pUC-PSIG-*ptrA* plasmid. A 6.2-kb DNA fragment containing the promoter, coding region, and terminator of *srdA* was amplified by PCR from the genomic DNA of *A. nidulans* A26 and inserted into pUC-PSIG-ptrA by the SLiCE reaction^53^ to obtain the pUC-PSIG-AN5849 plasmid. The DNA fragment used for *srdA* introduction was amplified from pUC-PSIG-AN5849 and introduced into *A. nidulans* SMGC-1 and SMGC-2 by the protoplast-PEG method^50^. The *srdA*-introduced SMGC-1 and SMGC-2 were designated as SMGC-1-PTSR and SMGC-2-PTSR, respectively. A DNA fragment containing the *ptrA* marker gene (but not *srdA*) was amplified by PCR from the plasmid pUC-PSIG-ptrA and introduced into SMGC-1 and -2, this served as the *srdA*-complementation control. The spontaneous mutants in which only the *prtA* marker gene was introduced were designated as SMGC-1-PT and SMGC-2-PT, respectively. The introduction of the fragments into the *srdA*-introduced and control strains was confirmed by Southern hybridization (Fig. S6C).

### Next-generation genomic sequencing of the suppressor mutants

The libraries for sequence analysis were prepared from the genomic DNA of SMGC-1, SMGC-2, and DRA, using the HiSeq SBS Kit v4 (Illumina, Inc., San Diego, USA). Genomic libraries were sequenced, using a HiSeq2500 (Illumina, Inc.), with a 100-base paired-end run. The reads were cleaned using Trimmomatic version 0.36^54^ and mapped to a reference genome using BWA version 0.7.17^55^. The total number of sample reads ranged between 22.8 and 26.9 M. In all samples, at least 92% of the bases were higher than Q30. The mapping rates of the samples ranged between 94.4 and 98.4%. The reference bases, covered at 50× depth of the samples, ranged between 90.6 and 96.6%. Using the mapping data, we carried out the variant calling of SMGC-1 and -2. The genomic sequence data of *A. nidulans* A4 were obtained from *Aspergillus* Genome Database^56^ and used as a reference genome for mapping cleaned reads. PCR duplicates in the mapping data were removed using Picared tools version 1.111 (http://picared.soucefoge.net/). The nucleotides altered in SMGC-1 and SMGC-2 were called using samtools, version 1.6^57^. The variants called by samtools were further filtered using bcftools version 1.6^58^. Finally, variants that met our filtering criteria (number of high-quality bases ≥ 10, genotype quantity ≥ 10, and allele frequency ≥ 95) were selected as the mutations found in SMGC-1 and SMGC-2.

### Observation of mutants using a scanning electron microscope

Fungal strains were cultivated on MMGp agar plates at 37 °C for 60 h. The samples were fixed overnight with 0.1 M cacodylate buffer (pH 7.4) containing 2% paraformaldehyde and 2% glutaraldehyde. The samples were further fixed with 0.1 M cacodylate buffer (pH 7.4) containing 1% tannic acid for 2 h. The samples were post-fixed with 0.1 M cacodylate buffer containing 2% osmium tetroxide for 3 h. All fixation steps were performed at 4 °C. The samples were dehydrated using an ethanol gradient and dried using the *tert*-butyl alcohol freeze-drying method^59^. Dried samples were coated with a thin layer of osmium using an osmium plasma coater. The conidiophores of the samples were observed under a scanning electron microscope, JSM-7500F (JEOL Ltd., Tokyo, Japan).

### Determination of conidiation efficiency

MMGp agar plates were inoculated with 5.0 × 10^3^ sample conidia, and incubated at 37 °C for 5 days. The diameters of five colonies were measured and an average diameter was calculated. The conidia-containing colonies were harvested in 12 mL of 0.05% Tween 20, using a spreader. The conidial suspensions were filtered through Miracloth (Merck Millipore, Billerica, Massachusetts, USA). The volume of the filtrate was adjusted to 12 mL by adding 0.05% Tween 20. The number of conidia in the filtrates was counted using a hemocytometer.

### Measurement of extracellular endo-xylanase production under SSC

Wheat bran was purchased from Yuutekku (Hokkaido, Japan) and prepared for SSC, as previously described^15^. Pre-moistened sterile wheat bran (10 g) was inoculated with 3.0 × 10^6^ conidia and incubated at 37 °C for 3 days with a relative humidity between 90 and 100%. The procedure for the preparation of crude extract from solid-state culture (SSC) has been previously described^15^. Azo-xylan (birch wood) (MEGAZYME, Wicklow, Ireland) was used as the assay substrate. The xylanase assay was performed according to the manufacturer’s instructions (Lot. 30601).

### Quantification of mycelia in solid-state cultures

The amount of mycelia in the SSCs was calculated as follows:

1 $$M\left( {SSC} \right) = Chi(SSC)/Chi(MYC),$$

where: *M(SSC)* = amount of mycelia in SSCs (mg/g dry SSCs), *Chi(SSC)* = chitin content in dry SSCs (μg/g dry SSCs), *Chi(MYC)* = chitin content in dry mycelia from liquid culture (μg/mg dry mycelia).

The detailed protocols for the quantification of chitin from dry SSCs, and dry mycelia from liquid culture, have been previously reported^15^.

### Determining growth sensitivities to cell wall perturbating agents

The growth sensitivity of the mutant strains to calcofluor white (CFW) was determined by conidia point inoculation of CFW-containing (2.5 μg/mL) MMGp agar plates. Five-fold dilution series (2.0 × 10^3^ to 2.5 × 10^5^) of conidial suspensions were spotted on the assay plate and incubated at 37 °C for 3 days. To determine growth sensitivity to caspofungin (CAS), 6.0 × 10^4^ conidia from the samples were point inoculated on CAS-containing (3.0 μg/mL) MMGp agar plates and incubated at 37 °C for 3 days. The sensitivities to these cell wall perturbating agents were determined by observing the phenotype of the colonies and their diameters.

### Western blot detection of phosphorylation levels of HogA and MpkA

Western blotting was used for analysis of phosphorylation of MAP kinases, HogA and MpkA. MMGp agar plate was covered with sterilized cellophane film. Sixty-thousand conidia were point-inoculated onto the cellophane film and incubated at 37 °C for 2 days. The fungal mycelia-containing cellophane film separated from the agar plate and rapidly frozen in liquid nitrogen. Preparation of crude extracts and their analysis by western blotting were performed as previously described^15,48^, using HogA and MpkA antibodies. The signal intensities of the western blots were quantified using ImageJ freeware (https://imagej.nih.gov/ij/).

### Statistical analyses

Statistical analyses (two-sample *t*-tests and multiple comparison tests) of the experimental data obtained in this study were carried out using R version 4.2.0 (https://cran.ism.ac.jp/). The *p*-values of the multiple comparison tests were adjusted by Holm’s method.

## Supplementary Information

Supplementary Information.

## Data Availability

The data are available upon request to the corresponding author.
