# Supplementary material for: srdA mutations suppress the rseA/cpsA deletion mutant conidiation defect in Aspergillus nidulans
Source: Sci Rep. 2023 Mar 15;13:4285. doi: 10.1038/s41598-023-31363-8 (PMC10017718; doi:10.1038/s41598-023-31363-8)
Supplement: Supplementary file 1 — Supplementary Information. [file 41598_2023_31363_MOESM1_ESM.pdf]

# Supplementary information

**[Title]**
***srdA* mutations suppress the *rseA*/*cpsA* deletion mutant conidiation defect in *Aspergillus nidulans***

**[Authors]**
**Masahiro Ogawa, Ryouichi Fukuda, Ryo Iwama, Yasuji Koyama & Hiroyuki Horiuchi**

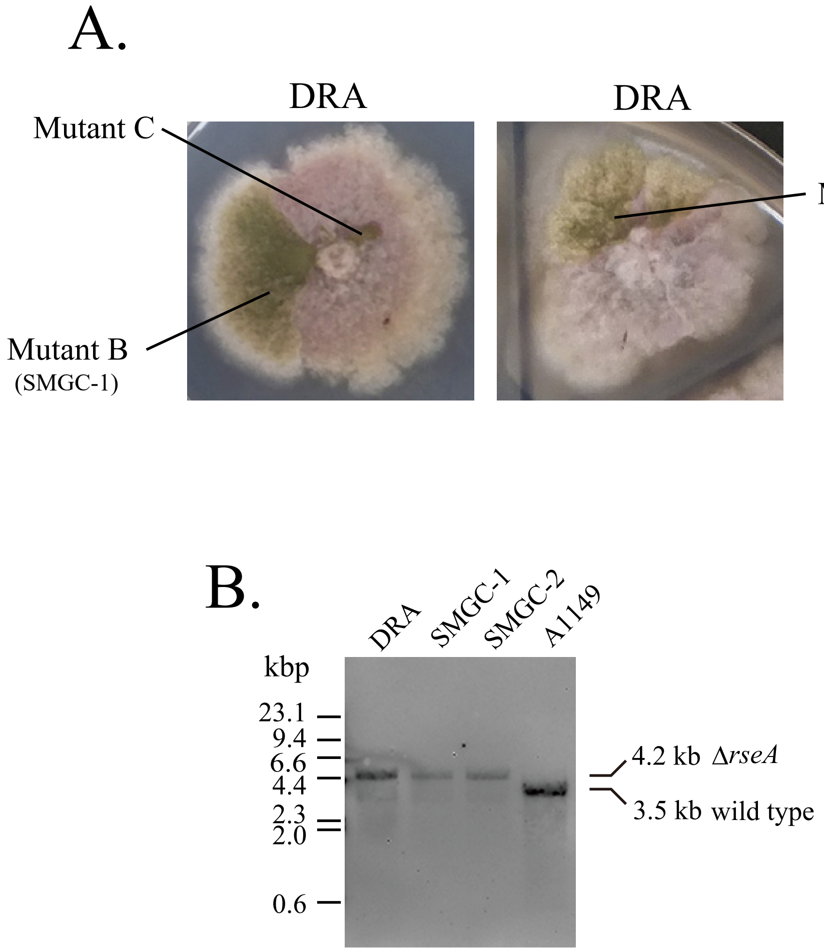


**Figure S1.** Suppression of the conidiation defect of the *rseA* deletion mutant (DRA) in spontaneous mutants. (**A**) Photographs of colonies with suppressor mutants (green-colored growth observed in mutants B, C, and D) of the *rseA* deletion mutant. DRA were cultivated on the MMGp agar plates at 37°C for seven days. (**B**) Southern hybridization confirmation of *rseA* deletions in SMGC-1 and SMGC-2. Strategy for the *rseA* deletion was previously reported[15]. The original data of Southern hybridization is shown in Figure S12.

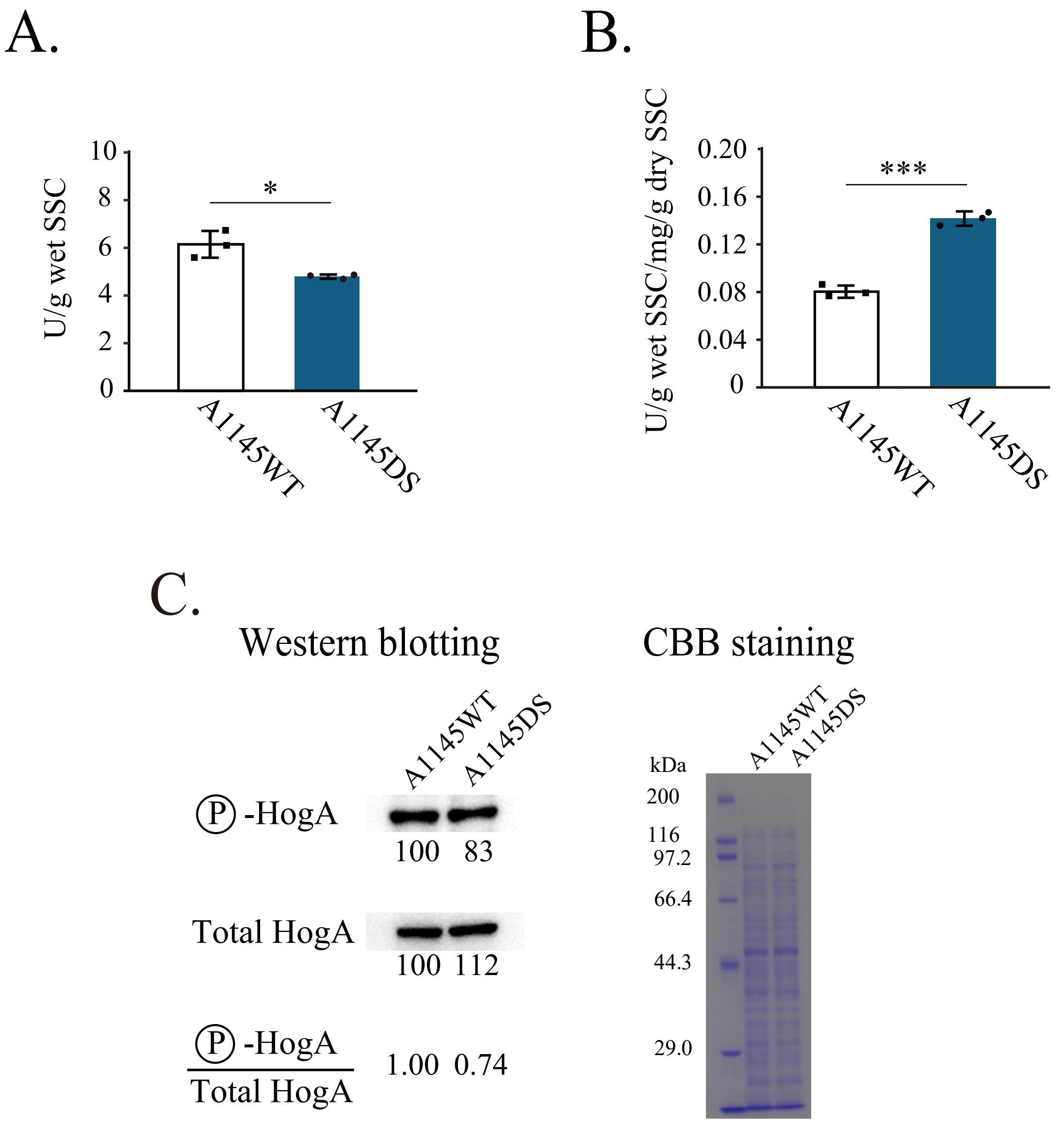


**Figure S2.** Extracellular endo-xylanase production and HogA phosphorylation of the Δ*srdA* mutant (A1145DS). (**A**) Endo-xylanase production of A1145DS and the wild-type strain (A1145WT) per g solid-state cultures (SSC). (**B**) Endo-xylanase production of A1145DS and A1145WT per mg mycelia. Experiments were conducted in triplicate. Bars indicate standard deviations. *: $p < 0.05$, and ***: $p < 0.001$ (Welch' s *t* test). (**C**) Western blotting of phosphorylated HogA and total HogA in A1145DS and A1145WT. Fifteen μg of strain crude extract was loaded onto SDS-PAGE gels. CBB staining of the samples was used as loading controls. The original data of western bolts and SDS-PAGE are shown in Figures S7, S8, and S11.

Identity of amino acid seauqnce (%)

| | *Paecilomyces variotii* | *Monascus purpureus* | *Penicillium rubens* | *Talaromyces marneffei* | *Blastomyces gilchristii* | *Emmonsia crescens* | *Helicocarpus griseus* | *Paracoccidioides lutzii* | *Coccidioides immitis* | *Uncinocarpus reesii* | *Trichophyton benhamiae* | *Nannizzia gypsea* | *Polytolypa hystricis* | *Alternaria tenuissima* | *Aureobasidium pullulans* | *Botrytis cinerea* | *Sclerotinia sclerotiorum* | *Fusarium oxysporum* | *Fusarium graminearum* | *Trichoderma reesei* | *Neurospora crassa* | *Pyricularia oryzae* |
|---|---|---|---|---|---|---|---|---|---|---|---|---|---|---|---|---|---|---|---|---|---|---|
| *Monascus purpureus* | 54 | | | | | | | | | | | | | | | | | | | | | |
| *Penicillium rubens* | 52 | 49 | | | | | | | | | | | | | | | | | | | | |
| *Talaromyces marneffei* | 46 | 42 | 42 | | | | | | | | | | | | | | | | | | | |
| *Blastomyces gilchristii* | 43 | 41 | 39 | 38 | | | | | | | | | | | | | | | | | | |
| *Emmonsia crescens* | 44 | 42 | 39 | 37 | 83 | | | | | | | | | | | | | | | | | |
| *Helicocarpus griseus* | 43 | 42 | 39 | 37 | 68 | 73 | | | | | | | | | | | | | | | | |
| *Paracoccidioides lutzii* | 42 | 40 | 39 | 37 | 67 | 69 | 65 | | | | | | | | | | | | | | | |
| *Coccidioides immitis* | 45 | 44 | 41 | 39 | 47 | 48 | 48 | 46 | | | | | | | | | | | | | | |
| *Uncinocarpus reesii* | 45 | 44 | 39 | 38 | 46 | 47 | 47 | 46 | 80 | | | | | | | | | | | | | |
| *Trichophyton benhamiae* | 46 | 44 | 42 | 38 | 48 | 49 | 48 | 47 | 58 | 57 | | | | | | | | | | | | |
| *Nannizzia gypsea* | 49 | 46 | 44 | 40 | 50 | 52 | 50 | 49 | 59 | 58 | 95 | | | | | | | | | | | |
| *Polytolypa hystricis* | 47 | 44 | 44 | 39 | 48 | 50 | 49 | 47 | 52 | 52 | 53 | 54 | | | | | | | | | | |
| *Alternaria tenuissima* | 34 | 33 | 31 | 30 | 31 | 31 | 32 | 32 | 34 | 32 | 33 | 33 | 32 | | | | | | | | | |
| *Aureobasidium pullulans* | 31 | 31 | 30 | 29 | 29 | 29 | 29 | 30 | 30 | 30 | 30 | 31 | 31 | 29 | | | | | | | | |
| *Botrytis cinerea* | 29 | 29 | 27 | 26 | 26 | 27 | 27 | 27 | 27 | 27 | 26 | 28 | 27 | 26 | 25 | | | | | | | |
| *Sclerotinia sclerotiorum* | 28 | 28 | 27 | 25 | 26 | 27 | 27 | 27 | 26 | 27 | 26 | 28 | 27 | 26 | 25 | 91 | | | | | | |
| *Fusarium oxysporum* | 27 | 27 | 24 | 25 | 26 | 26 | 27 | 25 | 25 | 26 | 25 | 26 | 27 | 24 | 24 | 40 | 39 | | | | | |
| *Fusarium graminearum* | 28 | 27 | 24 | 25 | 27 | 27 | 27 | 26 | 25 | 26 | 26 | 27 | 27 | 24 | 25 | 40 | 39 | 94 | | | | |
| *Trichoderma reesei* | 29 | 29 | 26 | 29 | 28 | 28 | 28 | 29 | 28 | 27 | 27 | 27 | 29 | 24 | 26 | 40 | 40 | 68 | 68 | | | |
| *Neurospora crassa* | 28 | 28 | 25 | 26 | 27 | 28 | 28 | 27 | 26 | 27 | 26 | 28 | 29 | 27 | 25 | 40 | 39 | 46 | 45 | 44 | | |
| *Pyricularia oryzae* | 27 | 28 | 25 | 25 | 26 | 26 | 27 | 27 | 27 | 27 | 26 | 28 | 26 | 25 | 26 | 32 | 32 | 34 | 34 | 37 | 35 | |
| *Aspergillus nidulans* | 50 | 48 | 47 | 41 | 38 | 39 | 39 | 40 | 40 | 39 | 41 | 43 | 41 | 32 | 31 | 27 | 27 | 25 | 25 | 27 | 25 | 25 |

**Figure S3.** Amino acid identity among the SrdA orthologs of 23 fungal species belonging to Ascomycota. NCBI RefSeq accession numbers or GenBank accession numbers of the SrdA orthologs are as follows: *P. variotii*, XP_028489531.1; *M. purpureus*, TQB68003.1; *P. rubens*, KAF3031016.1; *T. marneffei*, XP_002146493.1; *B. gilchristii*, XP_031580596.1; *E. crescens*, PGH33275.1; *H. griseus*, PGH06673.1; *P. lutzii*, XP_015701189.1; *C. immitis*, TPX26200.1; *U. reesii*, XP_002541351.1; *T. benhamiae*, XP_663453.1; *N. gypsea*, XP_003176777.1; *P. hystricis*, PGH15145.1; *A. tenuissima*, RYO03140.1; *A. pullulans*, THX04857.1; *B. cinerea*, XP_024550793.1; *S. sclerotiorum*, APA11859.1; *T. reesei*, XP_006966347.1; *F. oxysporum*, EWZ37522.1; *F. graminearum*, CAG2011157.1; *N. crassa*, XP_959206.3; *P. oryzae*, XP_003709896.1; and *A. nidulans*, XP_050467077.1.

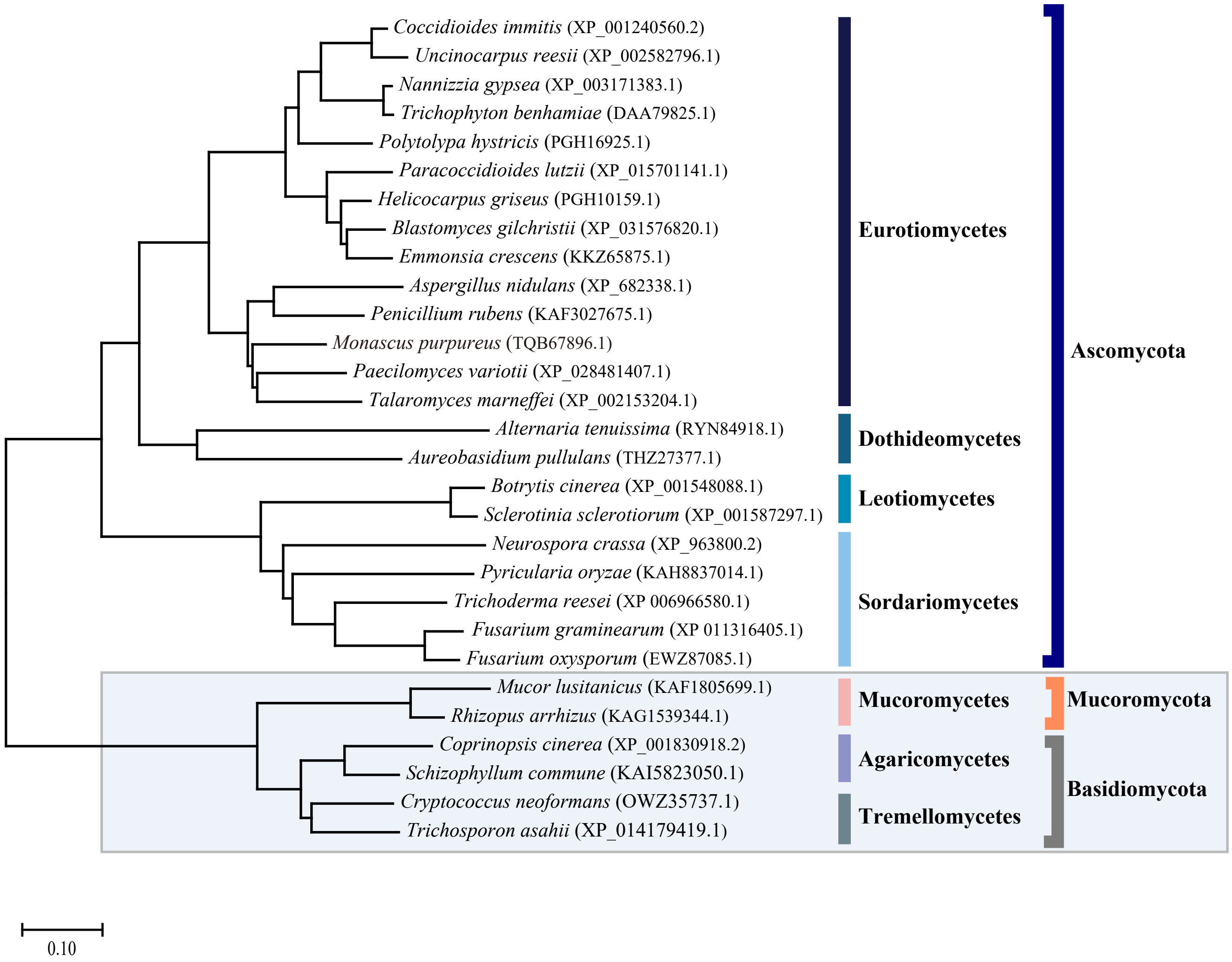


**Figure S4.** RseA/CpsA orthologs in 29 fungal species in Ascomycota, Basidiomycota, and Mucoromycota. A phylogenetic tree of RseA/CpsA orthologs in the 29 filamentous fungi. NCBI RefSeq accession numbers or GenBank accession numbers of the RseA/CpsA orthologs are indicated in parentheses. The phylogenetic tree was constructed using MEGA11[27]. Amino acid sequences of the RseA/CpsA orthologs were aligned using Clustal W algorithm[28]. Taxonomy of each fungal species was shown according to NCBI Taxonomy browser[29].

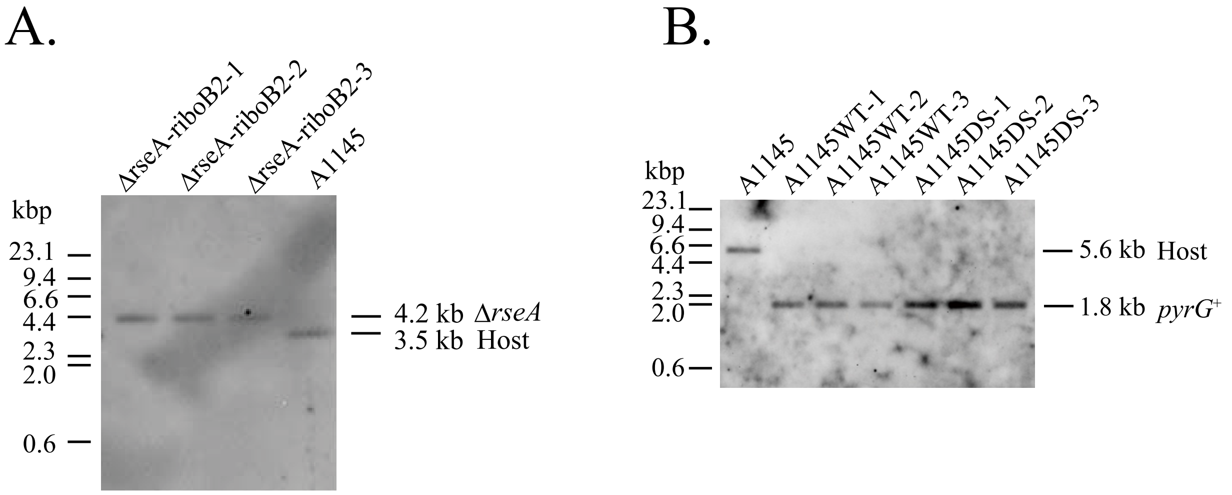


**Figure S5.** Southern hybridization confirming the *rseA* deletion and *pyrG* integration. (**A**) Southern hybridization of the wild type strain (A1145) and the Δ*rseA* strains (ΔrseA-riboB2-1–3). (**B**) Southern hybridization of the wild type strain (A1145) and confirmation of wild-type *pyrG* integration in the A1145WT-1–3 and A1145DS-1–3 strains. Strategy for the *rseA* deletion and the *pyrG* integration (insertion of *pyrG* at an upstream site of the *rseA* promoter) was performed as previously described[15]. The original data of Southern hybridization are shown in Figures S12 and S13.

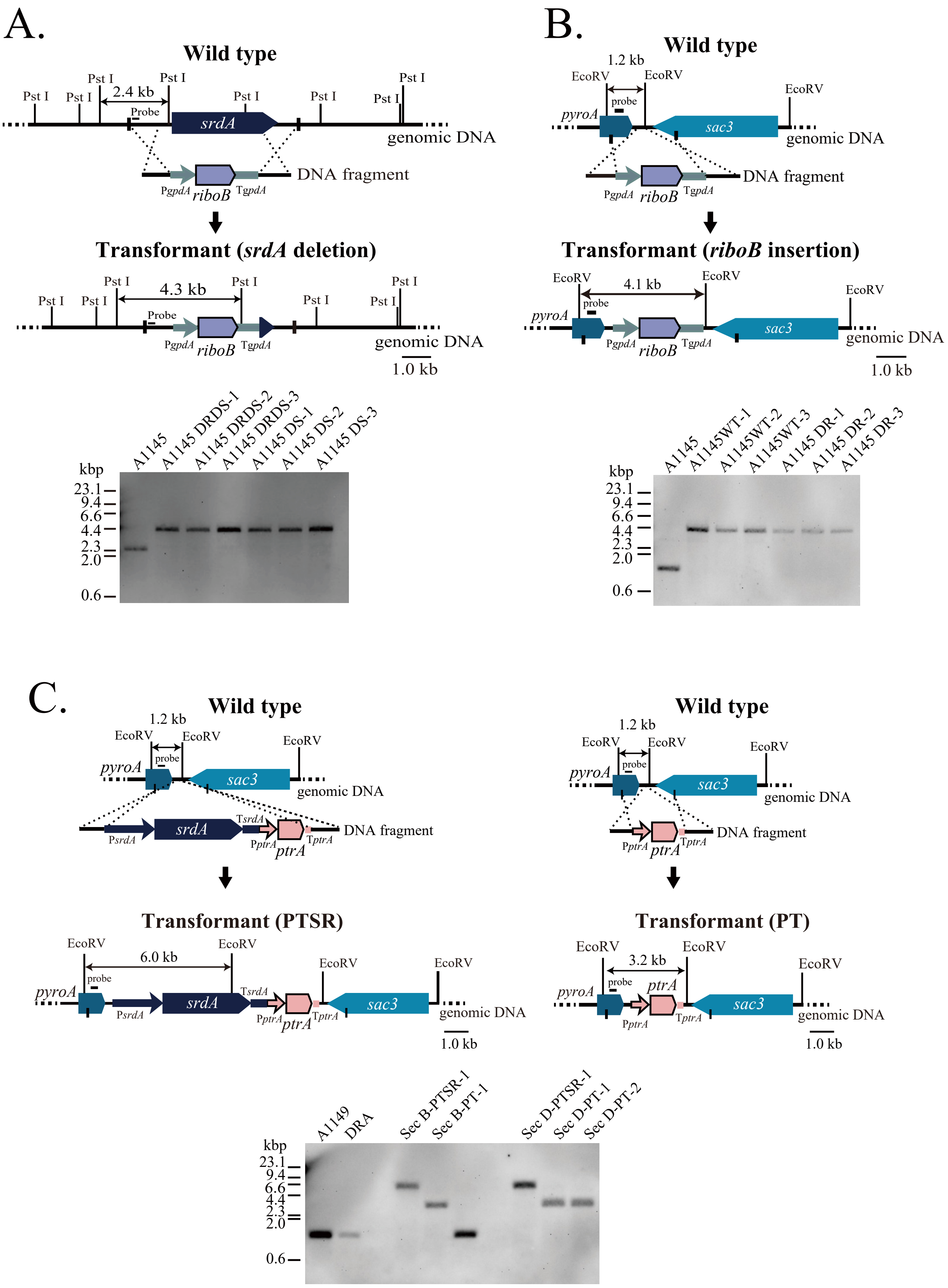


**Figure S6.** Southern hybridization confirming *srdA* deletions, *riboB* insertions, *srdA* integration, and *ptrA* integration. (**A**) *srdA* deletions in A1145DRDS and A1145DS. (**B**) *riboB* integrations in A1145WT and A1145DR. (**C**) *srdA* and *ptrA* integrations in SMGC-1 and SMGC-2 derivatives. The original data of Southern hybridization are shown in Figures S13 and S14.

# Phosphorylated HogA

## Membrane

Figure 5E Figure S2C

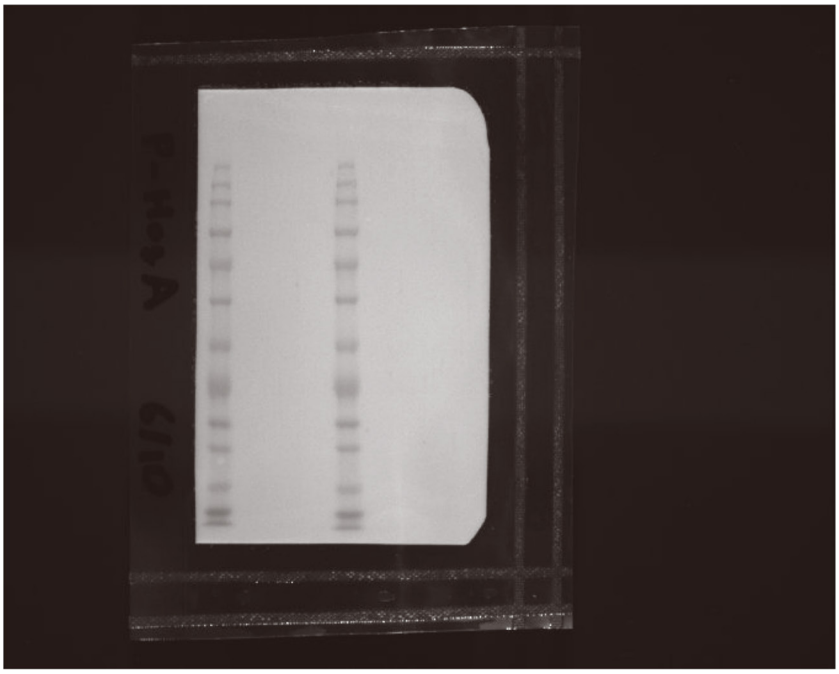

## Chemiluminescence

Figure 5E Figure S2C

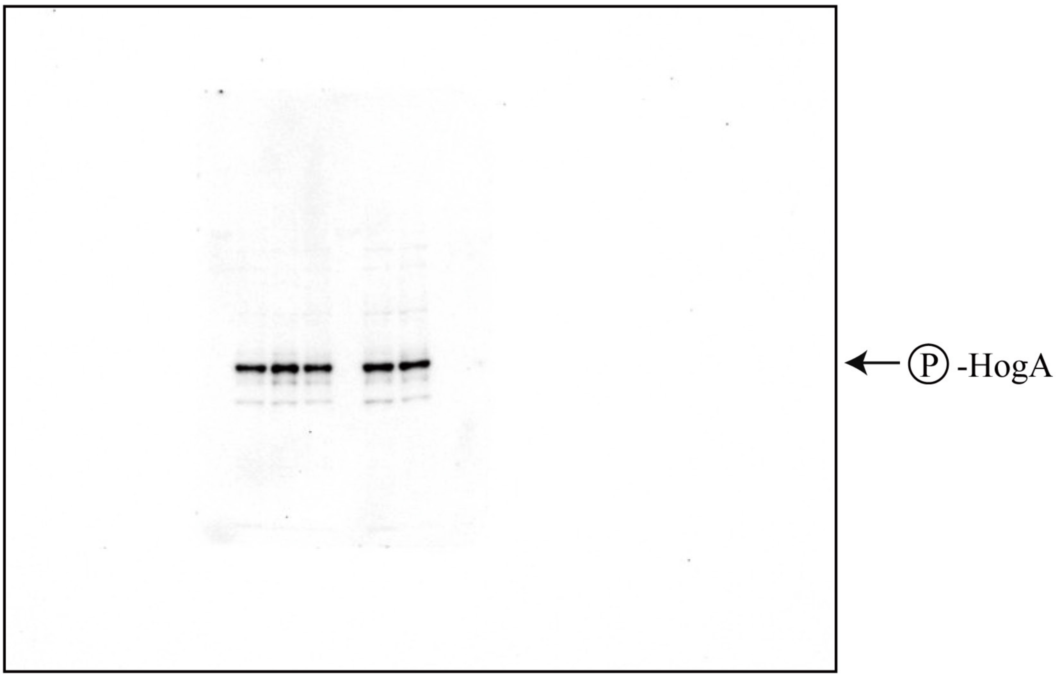


**Figure S7.** Detection of phosphorylated HogA by western blotting (the original data of Figure 5E and Figure S2C).

# Total HogA

## Membrane

Figure 5E Figure S2C

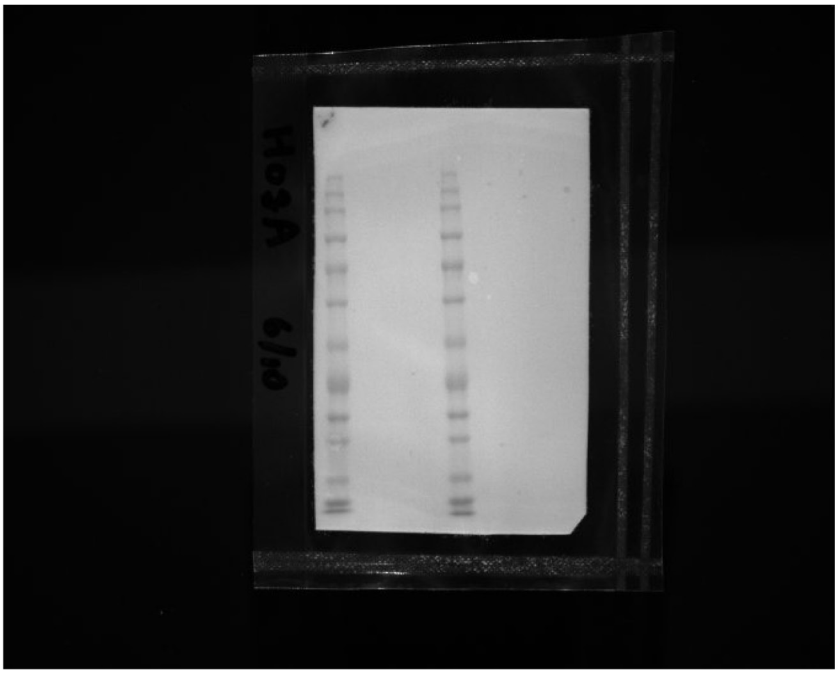


## Chemiluminescence

Figure 5E Figure S2C

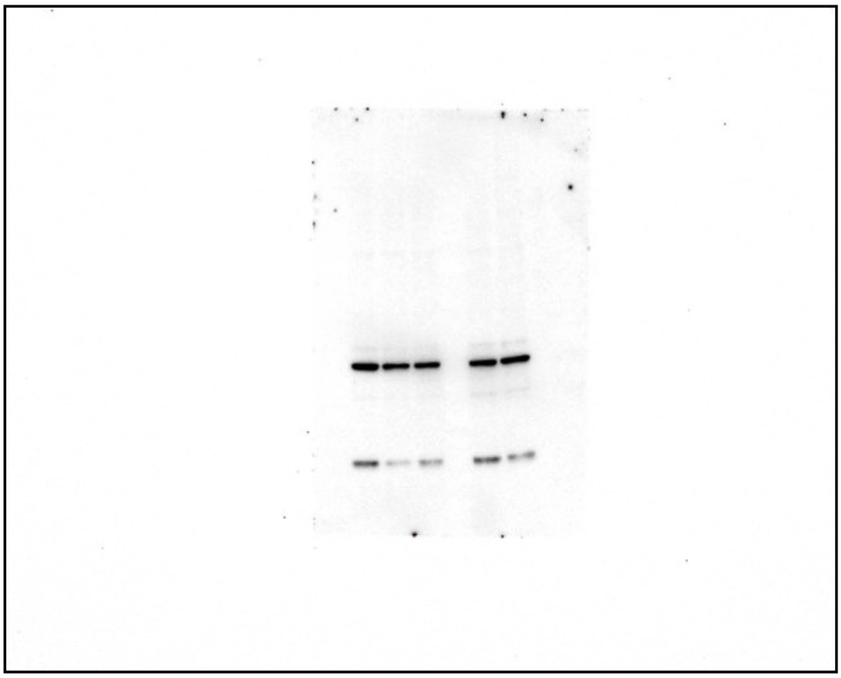

← Total HogA

**Figure S8.** Detection of total HogA by western blotting (the original data of Figure 5E and Figure S2C).

# Phosphorylated MpkA

## Membrane

Figure 5E

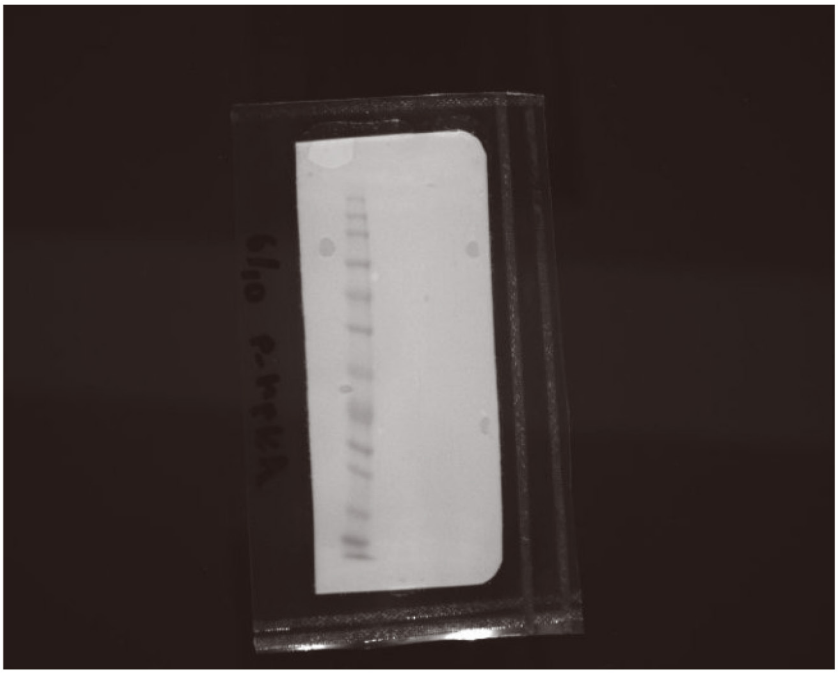

## Chemiluminescence

Figure 5E

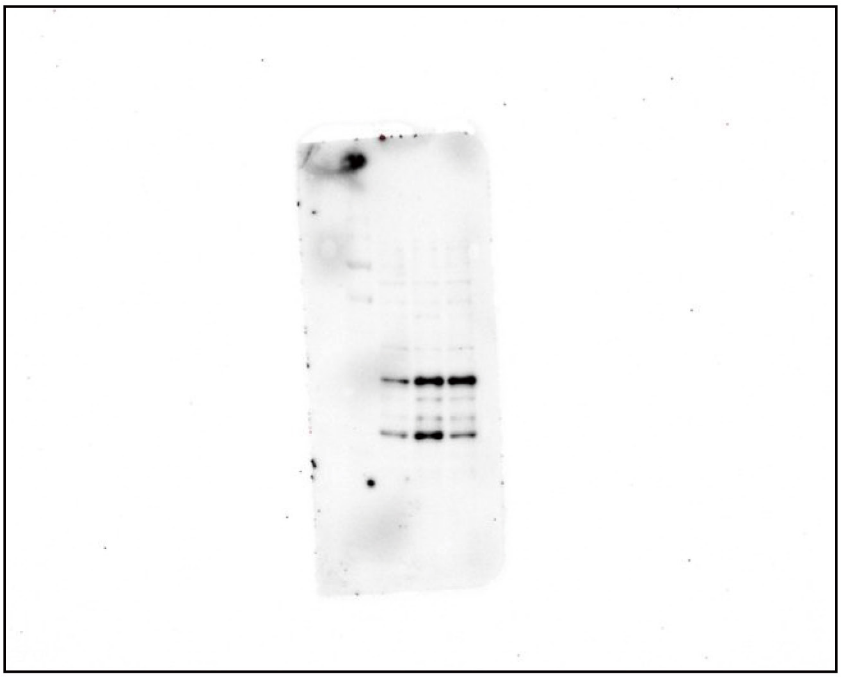


**Figure S9.** Detection of phosphorylated MpkA by western blotting (the original data of Figure 5E).

# Total MpkA

## Membrane

Figure 5E

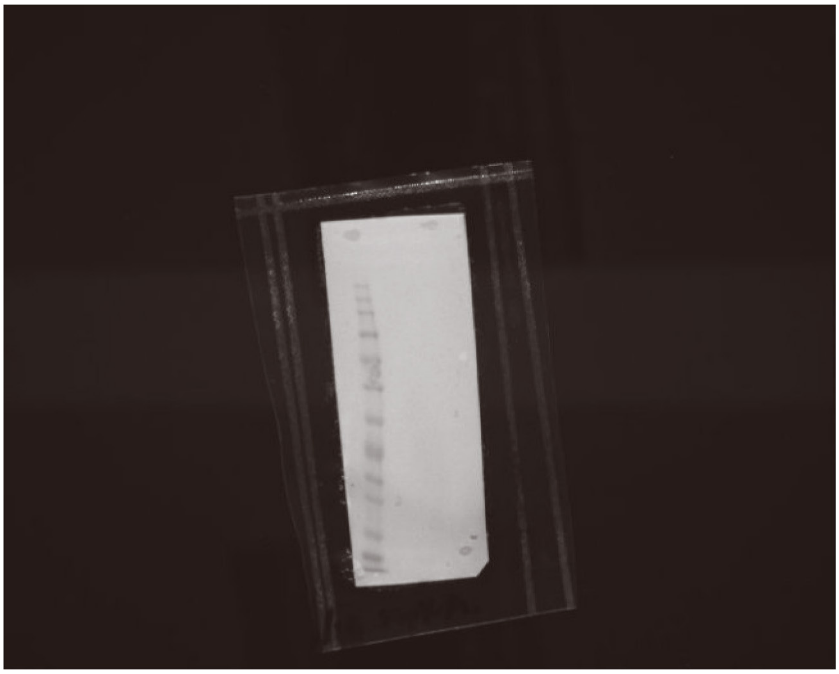

## Chemiluminescence

Figure 5E

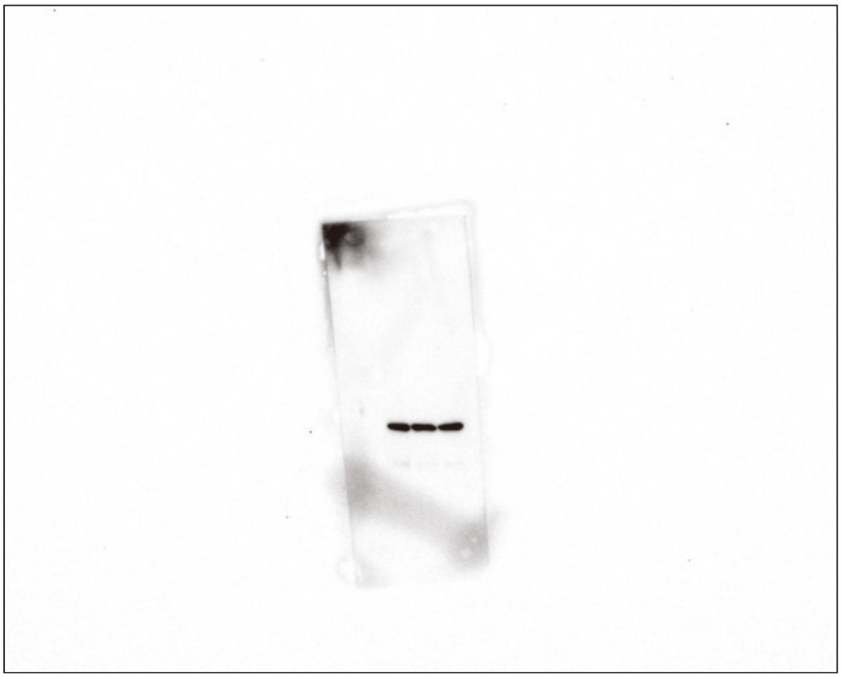


**Figure S10.** Detection of total MpkA by western blotting (the original data of Figure 5E).

# SDS-PAGE (CBB staining)

Figure 5E Figure S2C

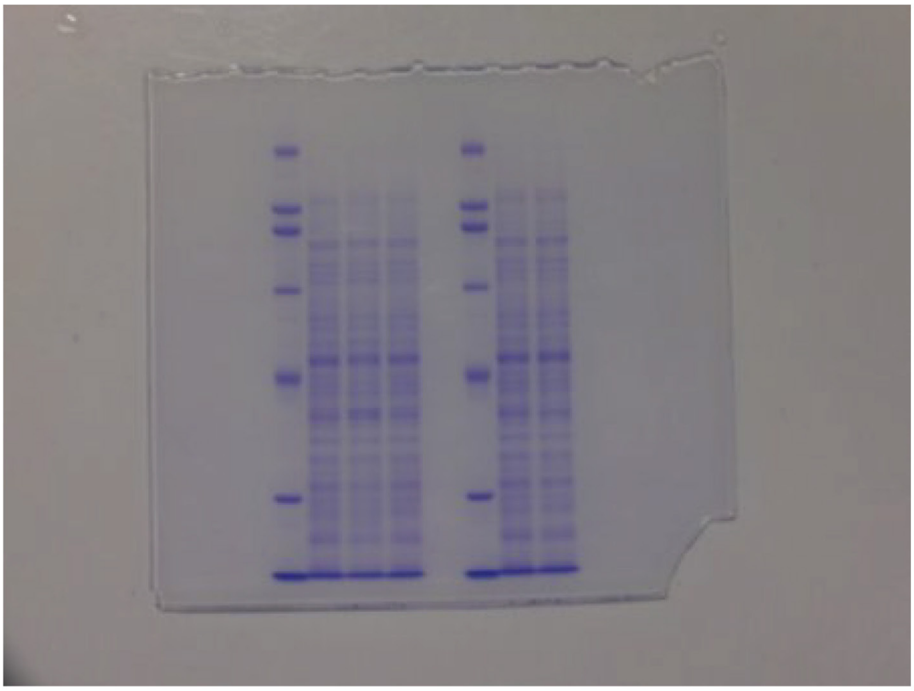

**Figure S11.** SDS-PAGE of the loading controls (the original data of Figure 5E and Figure S2C).

Chemiluminescence

Figure S1B

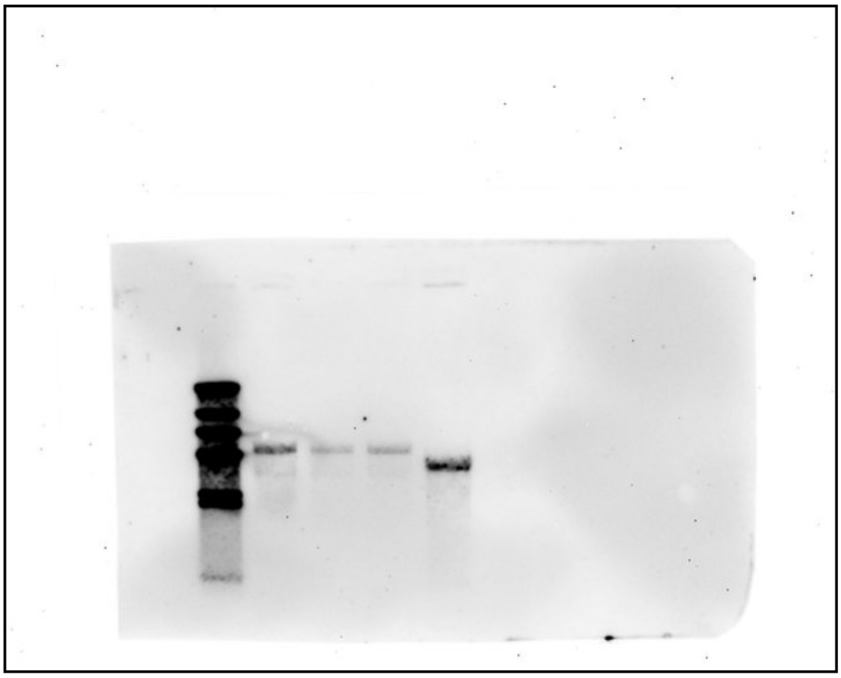

Chemiluminescence

Figure S5A

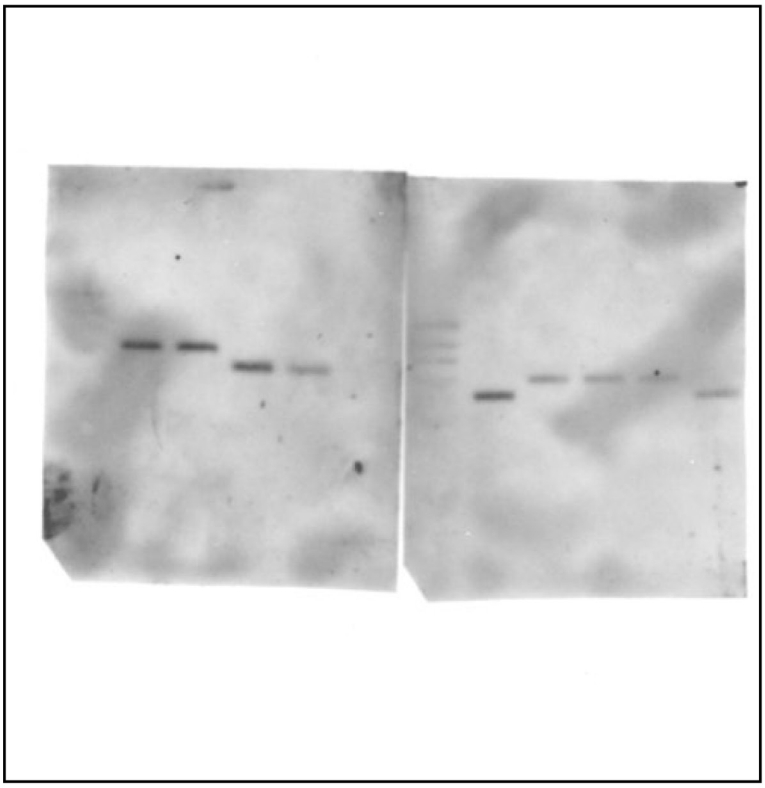

**Figure S12.** The original data of the Southern hybridization in Figures S1B and S5A.

Chemiluminescence

Figure S5B

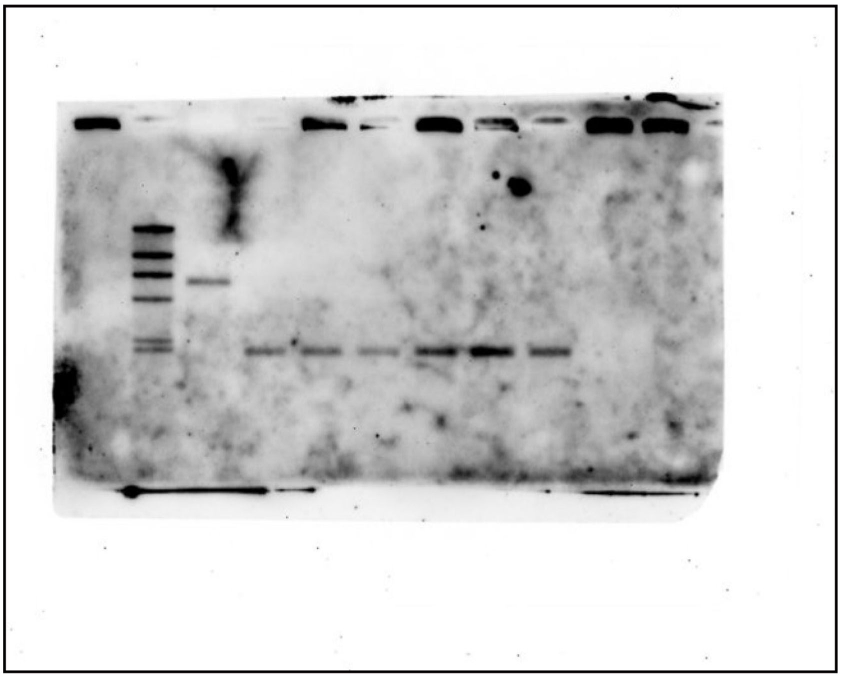

Chemiluminescence

Figure S6A

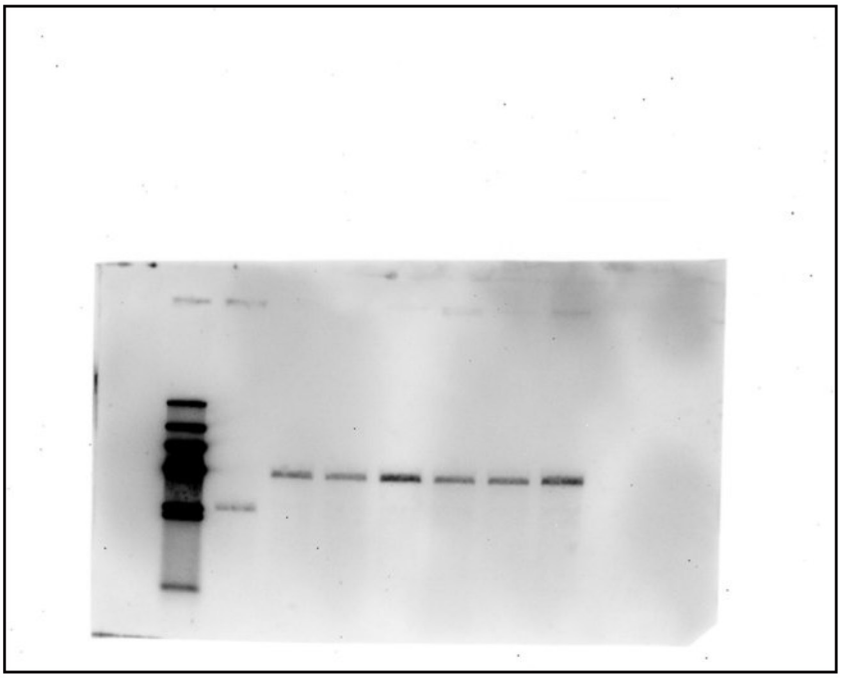

**Figure S13.** The original data of the Southern hybridization in Figures S5B and S6A.

Chemiluminescence

Figure S6B

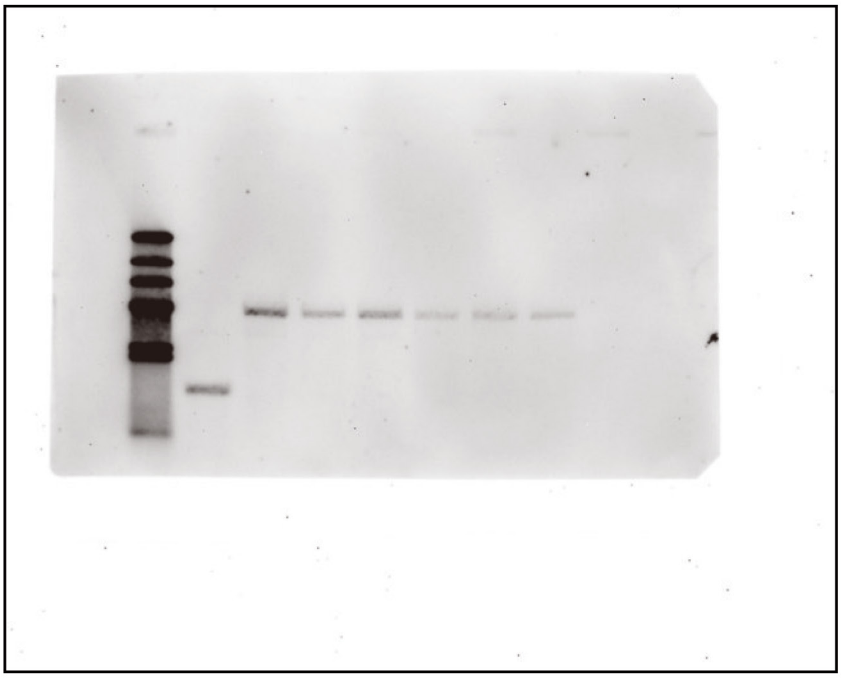

Chemiluminescence

Figure S6C

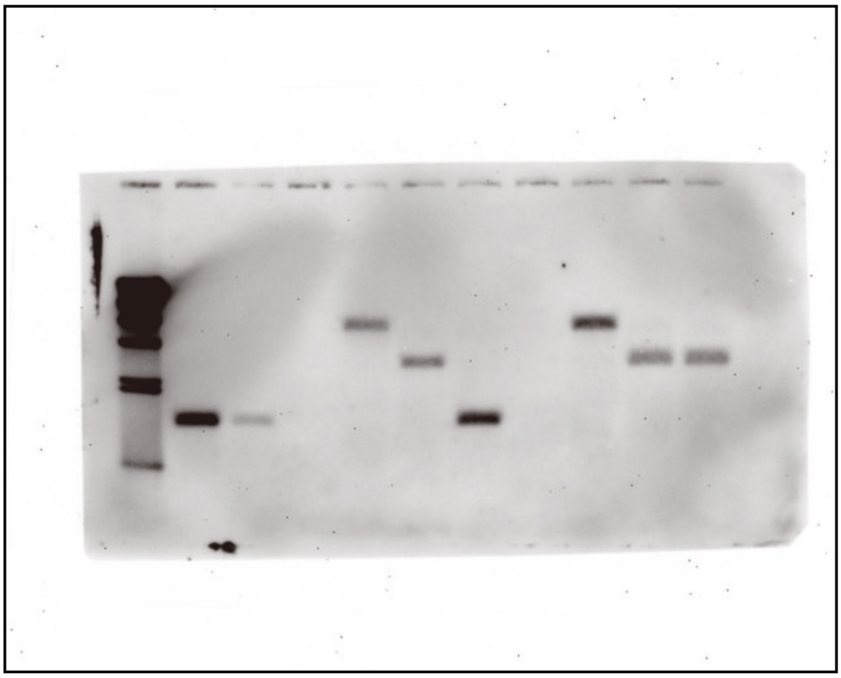

**Figure S14.** The original data of the Southern hybridization in Figures S6B and S6C.

# **Table S1.** Oligonucleotide primers used in this study

| Usage | Primer name | Sequence (5' to 3') | Description |
|---|---|---|---|
| Construction of pUC-PTgpdA-riboB | AN-PgpdA-F-SLiCE | GGATCCTCTAGAGTCCCAGACAGCTCTGGCGGCTCTGAGG | Amplification of *gpdA* promoter. Fusion PCR (Forward primer: AN-PgpdA-F-SLiCE). |
| | AN-PgpdA-R | GAGCGATATACAACCGTGTGATGTCTGCTCAAGCGGGGTAG | |
| | AN-riboB-F | TTGAGCAGACATCACACGGTTGTATATCGCTCTAGAATCCTCAC | Amplification of *riboB* ORF. |
| | AN-riboB-R | ACCTTGGAGATGTAGTCATTGAATCATATGGCCCATTTTAGAAATCTG | |
| | AN-TgpdA-F | CCATATGATTCAATGACTACATCTCCAAGGTTGATGCCCAATAG | Amplification of *gpdA* terminator. Fusion PCR (Reverse primer: AN-PgpdA-R-SLiCE). |
| | AN-TgpdA-R-SLiCE | GCATGCCTGCAGGTCTGAAACAATTGAGAAGACAGGAAGGGTCTG | |
| Constructions of pUC-AN5849 and pUC-AN 5849-DEL | AN5849-DEL-F-SLiCE | GGATCCTCTAGAGTCAAGAGATTTCGCGTCAAACCAATCAAATG | Cloning of *srdA* into pUC plasmid by SLiCE reaction. Amplification of the DNA fragment for *srdA* deletion. |
| | AN5849-DEL-R-SLiCE | GCATGCCTGCAGGTCCTTTTGCCAGGTTGATCAGGTTGATCAC | |
| | AN5849-DEL-vector-F | TCAATTGTTTCATGATATGACGCGCATTTCAAGTGACTTG | Amplification of a DNA fragment containing *srdA* used in construction of *srdA* deletion plasmid. |
| | AN5849-DEL-vector-R | GAGCTGTCTGGTGTAAGTGTAGCGTACCCGAGATGGTG | |
| | PgpdA-DEL-insert-F | GCTACACTTACACCAGACAGCTCTGGCGGCTCTGAGG | Amplification of *riboB* marker cassette used in construction of *srdA* deletion plasmid. |
| | TgpdA-DEL-insert-R | CGCGTCATATCATGAAACAATTGAGAAGACAGGAAGGGTCTG | |
| Confirmation of *srdA* deletion by PCR | 5849-DEL-F | CTGTATGATTGTCGCATGCGATAGACCG | Deletion of *srdA* region on the chromosomal DNA. |
| | 5849-DEL-R | CGGTCGTCATATGGTGAATGAAAGCTGC | |
| | AN5849-DEL-CF | AATCTGCCAATCTGCCAATCTGTAAATCAG | Insertion of the DNA fragment for *srdA* deletion on the chromosomal DNA (5'-side). |
| | 5849-5-Rv2 | TGTGATGTCTGCTCAAGCGGGGTAG | |
| | 5849-3-Fv2 | CAATAGGAAACAGGTCGGAAGCCAATGG | Insertion of the DNA fragment for *srdA* deletion on the chromosomal DNA (3'-side). |
| | 5849-3-Rv2 | GATAGCCAAAAACGAACCTTCTCGCGAGAG | |
| DIG-labeled probe for Δ*srdA* strain | AN5849-DEL-S1L-F | ATCGATTCGTGGCGGTTGGAGGCTGT | Preparation of the DIG labeled probe used in southern hybridization. |
| | AN5849-DEL-S1L-R | GAGTTCCTTAACTGCCAACCTACCAACC | |
| pUC-PSIG and pUC-PSIG-riboB | PSIG-L-F-SLiCE | GGATCCTCTAGAGTCCGTATTGGACACTTCGTTGAGTGCCAG | Amplification of the DNA fragments for homologus recombination (5'-side). Fusion PCR (Pyro3UTR-L-F-SLiCE). |
| | PSIG-L-R | CGCCAGAGCTGTCTGGAAGGTCCGTCAAAGCTAAGTAGAGCAGA | |
| | PSIG-PgpdA-F | AGCTTTGACGGACCTTCCAGACAGCTCTGGCGGCTCTGAGG | Amplification of *riboB* marker cassette. |
| | PSIG-TgpdA-R | TACTGGGCCACAGATGTGAAACAATTGAGAAGACAGGAAGGGTCTG | |
| | PSIG-R-F | CTTCTCAATTGTTTCACATCTGTGGCCCAGTAAAACTCTGTTTCC | Amplification of the DNA fragments for homologus recombination (3'-side). Fusion PCR (Pyro3UTR-R-R-SLiCE). |
| | PSIG-R-R-SLiCE | GCATGCCTGCAGGTCGGTCATTATAACCGGATGAACTGCTCTCG | |
| Construction of pUC-PISG-ptrA | PSIG-L-F-SLiCE | GGATCCTCTAGAGTCCGTATTGGACACTTCGTTGAGTGCCAG | Amplification of the intergenic region between *pyroA* and *sac3* (for homologus recombination, 5'-side). Fusion PCR (Pyro3UTR-L-F-SLiCE). |
| | PSIG-L-R-ptrA | GGATCCCGTAATCAATAAGGTCCGTCAAAGCTAAGTAGAGCAGA | |
| | PSIG-ptrA-F | AGCTTTGACGGACCTTATTGATTACGGGATCCCATTGGTAACG | Amplification of *ptrA* marker from pPTR I plasmid. |
| | PSIG-ptrA-R | TACTGGGCCACAGATGGACGATGAGCCGCTCTTGCATCTTT | |
| | PSIG-R-F-ptrA | AGAGCGGCTCATCGTCCATCTGTGGCCCAGTAAAACTCTGTTTCC | Amplification of the intergenic region between *pyroA* and *sac3* (for homologus recombination, 3'-side). Fusion PCR (Pyro3UTR-R-R-SLiCE). |
| | PSIG-R-R-SLiCE | GCATGCCTGCAGGTCGGTCATTATAACCGGATGAACTGCTCTCG | |
| Construction of pUC-PISG-AN5849 | ptrA-F-T5849 | AAAAGTGCTGGTGGAGATTGATTACGGGATCCCATTGGTAACG | Amplification of *ptrA* marker, and *pyroA* 3'-UTR resgion (for homologus recombination) used in construction of *srdA* complementation plasmid. |
| | PSIG-L-R-P5849 | CGACTCTAGCTAGTGTAAGGTCCGTCAAAGCTAAGTAGAGCAGA | |
| | P5849-F-PSIG | AGCTTTGACGGACCTTACACTAGCTAGAGTCGTCAGTGTACCG | Amplification of a DNA fragment containing *srdA* ORF, *srdA* promoter, and *srdA* terminator used in construction of *srdA* complementation plasmid. |
| | T5849-R-ptrA | GGATCCCGTAATCAATCTCCACCAGCACTTTTGGCCTATAACC | |
| Amplification of the DNA fragments used in fungal transformations | PSIG-L-F-ns1 | CGTATTGGACACTTCGTTGAGTGCCAG | *riboB* complementation, *srdA* complementation and *ptrA* insertion. |
| | PSIG-R-R-ns1 | GGTCATTATAACCGGATGAACTGCTCTCG | |
| Confirmation of the insertion of the DNA fragments by PCR | PSIG-L-F-C | CCGCCATGCTAATTGACCTGGATAGATC | Confirmation of DNA insertion at the intergenic region between *pyroA* and *sac3*. |
| | PSIG-R-R-C | TGTAGTGTCTAATGAAACCGAGGAAGACGC | |
| DIG-labeled probe for *srdA, riboB* complemented strains and *ptrA* inserted strain | PSIG-LS1-F | CGTTGAGTGCCAGGTTCGTCGTTTG | Preparation of DIG labeled probe used in southern hyblidization. |
| | PSIG-LS1-R | CTTTGAAGTTGTGCTTTGTGACGTGG | |

Additional sequences for fusion PCR were underlined.
Additional sequences for SLiCE reaction were double underlined.
